# Leveraging global genetics resources to enhance polygenic prediction across ancestrally diverse populations

**DOI:** 10.1016/j.xhgg.2025.100482

**Published:** 2025-07-18

**Authors:** Oliver Pain

**Affiliations:** 1 Maurice Wohl Clinical Neuroscience Institute, Department of Basic and Clinical Neuroscience, Institute of Psychiatry, Psychology and Neuroscience, King’s College London, London, UK

**Keywords:** polygenic scores, cross-ancestry prediction, GWAS, GenoPred

## Abstract

Genome-wide association studies (GWASs) from multiple ancestral populations are increasingly available, offering opportunities to improve the accuracy and equity of polygenic scores (PGSs). Several methods now aim to leverage multiple GWAS sources, but predictive performance and computational efficiency remain unclear, particularly when individual-level tuning data are unavailable. This study evaluates a comprehensive set of PGS methods across African (AFR), East Asian (EAS), and European (EUR) ancestries for 10 complex traits, using summary statistics from the Ugandan Genome Resource, Biobank Japan, UK Biobank, and the Million Veteran Program. Single-source PGSs were derived using methods including DBSLMM, lassosum, LDpred2, MegaPRS, pT + clump, PRS-CS, QuickPRS, and SBayesRC. Multi-source approaches included PRS-CSx, TL-PRS, X-Wing, and combinations of independently optimized single-source scores. All methods were restricted to HapMap3 variants and used linkage disequilibrium reference panels matching the GWAS super population. A key contribution is a novel application of the LEOPARD method to estimate optimal linear combinations of population-specific PGSs using only summary statistics. Analyses were implemented using the open-source GenoPred pipeline. In AFR and EAS populations, PGS combining ancestry-aligned and European GWASs outperformed single-source models. Linear combinations of independently optimized scores consistently outperformed current jointly optimized multi-source methods, while being substantially more computationally efficient. The LEOPARD extension offered a practical solution for tuning these combinations when only summary statistics were available, achieving performance comparable to tuning with individual-level data. These findings highlight a flexible and generalizable framework for multi-source PGS construction. The GenoPred pipeline supports more equitable, accurate, and accessible polygenic prediction.

## Teaser

This study benchmarks polygenic scoring methods across ancestrally diverse populations and introduces a summary-statistic-based strategy for optimally combining population-specific scores. The findings support flexible, efficient, and equitable genomic prediction using multi-ancestry GWASs, with all methods implemented in the open-source GenoPred pipeline.

## Introduction

Genome-wide association studies (GWASs) have identified thousands of genetic variants associated with a range of outcomes.^1^^,^^2^ Polygenic scores (PGSs) harness this wealth of data by aggregating the effects of numerous genetic variants to estimate an individual’s genetic predisposition to specific traits or diseases.^3^ As GWAS sample sizes continue to grow and novel PGS methods emerge, the predictive power and utility of PGSs in research and clinical settings have significantly improved. Of particular interest is the use of PGSs for disease risk stratification,^4^^,^^5^ which creates opportunities to enhance disease prevention, early diagnosis, and targeted treatment.^6^^,^^7^^,^^8^

However, a major limitation of current GWASs is the overrepresentation of individuals of European (EUR) ancestry, which leads to a substantial decrease in PGS performance across populations.^9^^,^^10^^,^^11^^,^^12^ PGSs derived from EUR-based GWASs typically perform better in EUR populations than in non-EUR populations, such as those of African (AFR) or East Asian (EAS) ancestry. This disparity reduces the clinical utility of PGSs, and their current use would exacerbate health inequalities. The increasing availability of GWASs within non-EUR populations has prompted the development of PGS methodology that can leverage multiple population-specific GWASs,^13^ aiming to improve predictive performance and ensure broader applicability in diverse populations.

**Figure 1:**
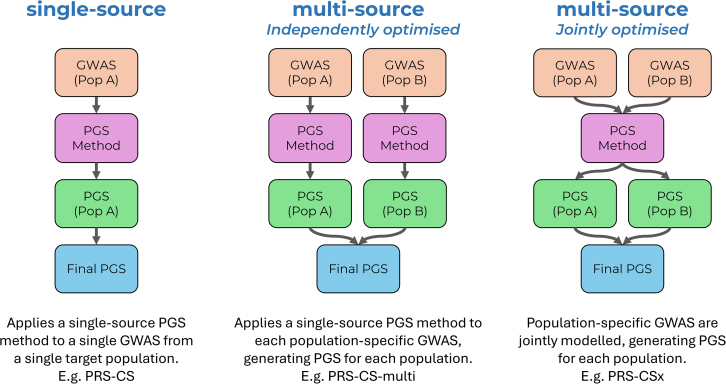
Overview of PGS methods leveraging GWASs from one or more populations Pop, population.

PGSs are typically calculated by aggregating genetic variants across the genome, weighting each variant according to how strongly it relates to a specific trait. Various PGS methods exist for estimating these variant weights based on GWAS results. PGS methods can be broadly categorised as *single-source* or *multi-source* (Figure 1). Single-source PGS methods, such as PRS-CS, derive the variant weights from a single GWAS.^14^ Multi-source methods, in contrast, incorporate multiple GWASs from different populations to improve prediction. *Independently optimized* multi-source methods apply single-source PGS methods separately to each GWAS, producing multiple ancestry-specific PGSs that are later linearly combined to optimize prediction for a given target population. These are typically denoted by appending “-multi” to the base method (e.g., PRS-CS-multi). More recently, *jointly optimized* multi-source methods have been developed, such as PRS-CSx, which leverage cross-population data to enhance effect size estimation and improve robustness.^15^^,^^16^^,^^17^ Like independently optimized methods, they produce separate PGSs for each population, which can also be linearly combined to refine prediction.

Previous research has shown that integrating multiple GWASs from different populations enhances PGS accuracy, but the optimal approach remains uncertain. Several studies suggest that jointly optimized PGS methods outperform independently optimized approaches.^15^^,^^17^^,^^18^ However, one recent study indicates that independently optimized methods can surpass currently available jointly optimized strategies.^19^ This is likely because advances in single-source methodologies have not yet been incorporated into jointly optimized multi-source methods. Additionally, independently optimized methods are reported to require fewer computational resources,^19^ making them more practical for large-scale applications. A comprehensive and independent evaluation of currently available multi-source PGS strategies is needed to determine the most effective approach.

An important consideration when comparing PGS methods is whether they can be tuned using only GWAS summary statistics. This approach, often termed “pseudovalidation” or “auto” modeling, allows tuning of the final PGS without needing individual-level genotype or phenotype data. Most PGS methods generate multiple scores by varying hyperparameters, such as *p*-value thresholds or shrinkage parameters. Multi-source methods generate separate PGSs for each ancestry-specific GWAS. To improve prediction in a given target population, these scores can be combined linearly to find an optimal weighted sum. Determining the best weights, however, requires an additional tuning step. While individual-level data can be used for this tuning, it is often inaccessible. Alternative techniques (such as sample-splitting to avoid overfitting) introduce additional complexity. In contrast, approaches that rely solely on GWAS summary statistics for tuning offer a more practical solution. However, while a range of summary-statistic tuning methods exist for optimizing individual PGS hyperparameters, fewer approaches are available for tuning the optimal linear combination of population-specific PGSs. Notably, no summary-statistic approach has been tested for independently optimized multi-source methods. One promising solution is the LEOPARD method, which estimates the optimal linear combination of population-specific PGSs without requiring individual-level data. Currently, LEOPARD is used only within the X-Wing multi-source PGS method and has not yet been explored for other PGS methods.^17^ Extending this approach to other multi-source methods could provide a generalizable strategy for tuning the optimal linear combination of population-specific PGSs. Addressing this gap is essential for enhancing the robustness and applicability of multi-source PGS approaches.

The GenoPred pipeline is an easy-to-use, high-performance, reference-standardized, and reproducible workflow for polygenic scoring.^20^ The GenoPred reference-standardized framework has been used in previous studies for comparing polygenic scoring methods.^21^ The GenoPred pipeline makes the identified leading approaches more accessible by facilitating their implementation. Although earlier versions of GenoPred supported GWAS and target samples from various ancestral populations, they were limited to single-source PGS methods, restricting the pipeline’s ability to fully leverage the increasingly available ancestrally diverse GWAS data.

In this study, the GenoPred pipeline is used to systematically evaluate the performance of various PGS methods and modeling approaches across African (AFR), East Asian (EAS), and European (EUR) populations. Furthermore, this study introduces a novel application of the LEOPARD method as a generalizable and efficient solution for estimating the optimal linear combination of population-specific PGSs. The aim is to optimize and broaden access to multi-source PGS methods that leverage ancestrally diverse datasets, contributing to more equitable, accurate, and accessible polygenic prediction.

## Materials and methods

### Overview

**Figure 2:**
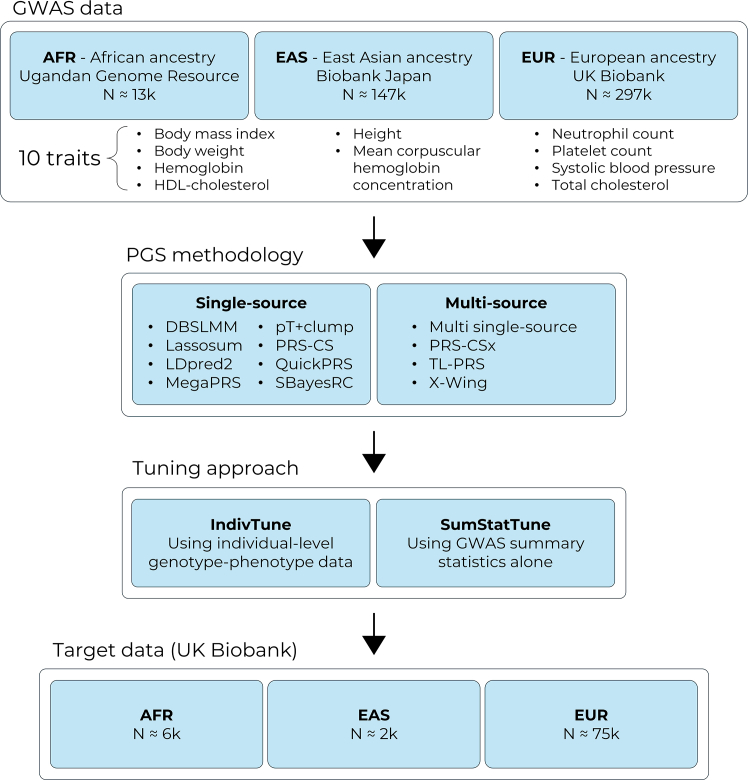
Study design overview “N” indicates median sample size.

This study evaluated the performance of PGS methods and modeling approaches across diverse populations using GWAS and target datasets of EUR, EAS, and AFR ancestry (Figure 2). EUR GWAS summary statistics were generated within a training subset of European ancestry individuals in the UK Biobank sample (UKB).^22^ Publicly available EAS and AFR GWASs were obtained from Biobank Japan (BBJ)^23^ and the Ugandan Genome Resource (UGR),^24^ respectively. An independent target sample of EUR, EAS, and AFR ancestry individuals within the UKB were used to evaluate the predictive performance of the PGS. To compare PGS methods, 10 quantitative traits available in the UKB, BBJ, and UGR were selected to represent a range of genetic architectures. Selected traits include body mass index, body weight, hemoglobin, high-density lipoprotein (HDL)-cholesterol, height, mean corpuscular hemoglobin concentration, neutrophil count, platelet count, systolic blood pressure, and total cholesterol. In addition to the main analysis, sensitivity analyses were performed using larger AFR ancestry GWASs from the Million Veteran Program (MVP) sample^25^ and downsampled EUR GWASs from UKB to assess the robustness of findings to GWAS sample size.

### UK biobank

UKB is a prospective cohort study that recruited >500,000 individuals aged between 40 and 69 years across the United Kingdom.^22^ The UKB received ethical approval from the North West-Haydock Research Ethics Committee (ref. 16/NW/0274).

UKB was used to generate GWAS summary statistics for EUR ancestry and to evaluate the predictive utility of PGS in individuals of EUR, EAS, and AFR ancestry. To avoid sample overlap between the EUR GWAS and EUR target samples, EUR individuals in UKB were split into a training subset for GWAS (80%) and testing subset for evaluating PGS (20%).

#### Ancestry inference

The ancestry of UKB individuals was genetically inferred, matching individuals to populations within the reference genetic dataset, a combination of samples from 1000 Genomes phase 3 and the Human Genome Diversity Project (1KG + HGDP).^26^^,^^27^ Outlier individuals were then removed from each inferred population. Ancestry inference and outlier detection was performed using the GenoPred pipeline,^20^ using the imputed UKB genetic data as input in PLINK2 binary format (.pgen)^28^ and filtered to include variants with a minor allele frequency of ≥1% and an imputation INFO score >0.4.

In brief, GenoPred estimates each individual’s probability of belonging to a reference population using a multinomial elastic net model trained on six genetic principal components derived from the reference dataset. These components are projected onto UKB, and individuals are assigned to a population if the predicted probability is >0.95. For outlier detection, GenoPred uses principal-component analysis within each inferred UKB population to capture population-specific structure and batch effects, identifying outliers based on k-means clustering centroids. Full details are available in the GenoPred documentation.^20^

#### Outcome trait preparation

Outcome trait data were extracted using the ukbkings R package.^29^ Unrelated individuals were identified via the UKB-provided kinship matrix and the GreedyRelated software v1.2.1 (see data and code availability). These individuals were then split into their inferred ancestral populations. EUR individuals were further split into training (80%) and testing subsets (20%). Within each group, the outcome was inverse rank-based normalized, and then covariates were regressed out.^30^ Covariates included age, sex, and the first 20 within-UKB genetic principal components (PCs). The residuals were then scaled and centered to have a mean of 0 and standard deviation of 1.

### GWAS summary statistics

For the main analysis, GWAS summary statistics for EUR, EAS, and AFR populations were obtained from UKB, BBJ, and UGR, respectively.^23^^,^^24^ Publicly available BBJ and UGR GWAS summary statistics were downloaded for this study. GWAS summary statistics for EUR were derived in the training subset of UKB for this study using PLINK2.^28^

A set of 28 metabolic, cardiovascular, and anthropometric traits were identified as being available across UKB, BBJ, and UGR. Descriptive statistics for these traits are provided in Table S1 (including BBJ/UGR download links and UKB field codes).

Quality control of GWAS summary statistics was performed using the GenoPred pipeline. In brief: strand-ambiguous variants are removed; variants are aligned to the 1KG + HGDP reference (restricted to HapMap3 variants; see data and code availability); and results were filtered based on imputation quality (INFO <0.9), minor allele frequency (MAF <0.01 or MAF discrepancies with reference >0.2), valid *p* values (0 < *p* ≤ 1), presence of unique SNP IDs, and acceptable sample size ranges. Missing BETA coefficients and standard errors (SEs) are calculated if absent. Full details are available in the GenoPred documentation.^20^

To better characterize the ancestry composition of each GWAS sample, reference PCs derived from the 1KG + HGDP panel were projected into each GWAS using allele frequency data, allowing visual comparison of ancestry differences between the GWAS and reference populations. In addition, ancestry proportions were estimated using the snp_ancestry_summary function from the bigsnpr R package^31^ (see data and code availability), which leverages allele frequency profiles and precomputed projections. This ancestry composition analysis was conducted separately and is not currently part of the standard GWAS quality control procedures implemented in the GenoPred pipeline.

### Selecting traits for comparison

To minimize computational costs associated with running all PGS methods repeatedly, a subset of 10 traits was selected for downstream analyses. These traits were chosen to represent a range of genetic architectures, including SNP-based heritability (SNP-*h*^2^) and polygenicity. LD score regression (LDSC) with GWAS summary statistics was used to estimate SNP-*h*^2^.^32^ The AVENGEME software (Additive Variance Explained and Number of Genetic Effects Method of Estimation) was used to estimate SNP-*h*^2^ and the proportion of variants with no effect on the trait (pi0), a metric representing the inverse of polygenicity.^33^ AVENGEME uses PGS associations across a range of *p*-value thresholds to estimate these parameters. The GenoPred pipeline was used to calculate the PGS in UKB using the *p*-value thresholding and clumping (*p*T + clump) method. Association analysis was then conducted in R version 4.2.3.^34^

Traits were initially filtered to retain those with a positive SNP-*h*^2^ point estimate from both LDSC and AVENGEME in EUR, EAS, and AFR populations. The SNP-*h*^2^ and pi0 estimates from AVENGEME (using EUR data) were then used to randomly select 10 traits that capture a range of these values, and therefore a range of genetic architectures. Specifically, both heritability and polygenicity estimates were divided into five bins based on their observed distribution. For SNP-*h*^2^, the bins were (0.052–0.090), (0.090–0.129), (0.129–0.167), (0.167–0.205), and (0.205–0.244). For pi0, the bins were (0.854–0.878), (0.878–0.901), (0.901–0.924), (0.924–0.947), and (0.947–0.971). Traits were grouped by the unique combinations of these bin assignments, and one trait was randomly selected from each group. If more than 10 traits were selected, a random subset of 10 was retained. EUR AVENGEME results were used as the estimates were most accurate due to the larger sample size compared with EAS and AFR populations. The selected traits are indicated in Table S1.

### PGS methodology

A range of leading summary-statistic PGS methods, including single- and multi-source, were applied using the GenoPred pipeline. Single-source methods include DBSLMM,^35^ lassosum,^36^ LDpred2,^37^ MegaPRS,^38^ pT + clump,^28^ PRS-CS,^14^ QuickPRS (Fast variant of MegaPRS, see data and code availability), and SBayesRC.^19^ Each of these methods was also evaluated as independently optimized multi-source methods. This involves applying the single-source PGS method to each GWAS and linearly combining the population-specific PGS to optimize prediction in a given target population. Jointly optimized multi-source methods include PRS-CSx^15^ and X-Wing.^17^ In contrast to these, the TL-PRS method represents a distinct multi-source approach: it starts with an existing “baseline” PGS model (a set of SNP weights tuned in one population) and further tunes it using GWAS data from the target population’s ancestry. TL-PRS is a post hoc adjustment applied to an already-developed PGS, rather than a method that simultaneously derives weights from multiple GWASs.

These methods were selected based on their performance in previous literature. The multi-source method BridgePRS^39^ was not evaluated in this study because it currently cannot produce score files without access to individual-level phenotype and genotype data. Although several other PGS methods require individual-level data to tune the final PGS, they produce a series or score files without the need for individual-level training data, in keeping with the reference-standardized framework of the GenoPred pipeline.

As noted, the TL-PRS method is distinct from other multi-source methods; therefore, TL-PRS was evaluated separately from other methods. The originally proposed workflow for TL-PRS involves two tuning steps: first tuning a baseline PGS model to select hyperparameters, and then further tuning that model using the target population’s GWAS to select the learning rate (gradient). For simplicity, this study’s evaluation used baseline PGS models that were tuned using summary statistics alone. This meant that individual-level data were required only for the second step of tuning the baseline PGS with the target population’s GWAS. When GWAS summary statistics are available for two populations, TL-PRS can be run in both directions (a procedure referred to as MTL-PRS). In other words, one can tune a PGS from population A using population B’s GWAS, and vice versa, then linearly combine the two population-specific PGSs. PGSs adjusted using TL-PRS are denoted by appending “TL-” to the method’s name (e.g., TL-SBayesRC and TL-SBayesRC-multi). A schematic representation of the TL-PRS workflow is shown in Figure S1.

The same 1KG + HGDP reference data were used for all PGS methods except PRS-CS, PRS-CSx, and X-Wing. For those three methods, the 1000 Genomes reference data provided with their software were used, as it is not straightforward to create a custom reference in the required format. As a sensitivity analysis, a collection of methods were run using only 1KG reference individuals to assess the impact of different reference data.

It should be noted that SBayesRC was run using the recommended “Baseline model 2.2” functional annotations, and MegaPRS and QuickPRS were run using the recommended “BLD-LDAK” functional annotations. Other PGS methods do not consider functional annotations.

### Estimating optimal linear combination of population-specific PGS

As mentioned above, no summary-statistic approach has been tested for tuning the linear combination of population-specific PGS from independently optimized multi-source methods. Among the jointly optimized multi-source methods, each method provides a summary-statistic-based tuning approach. PRS-CSx has an option (--meta) to combine population-specific posterior SNP effect sizes using inverse-variance meta-analysis, based on PGSs generated with the “auto” phi model.^15^ In contrast, X-Wing employs the LEOPARD method,^17^ a more advanced approach that estimates the optimal linear combination of population-specific PGS for a given target population, allowing greater flexibility when certain GWASs are more relevant than others. There is no summary-statistic tuning approach for TL-PRS.

**Figure 3:**
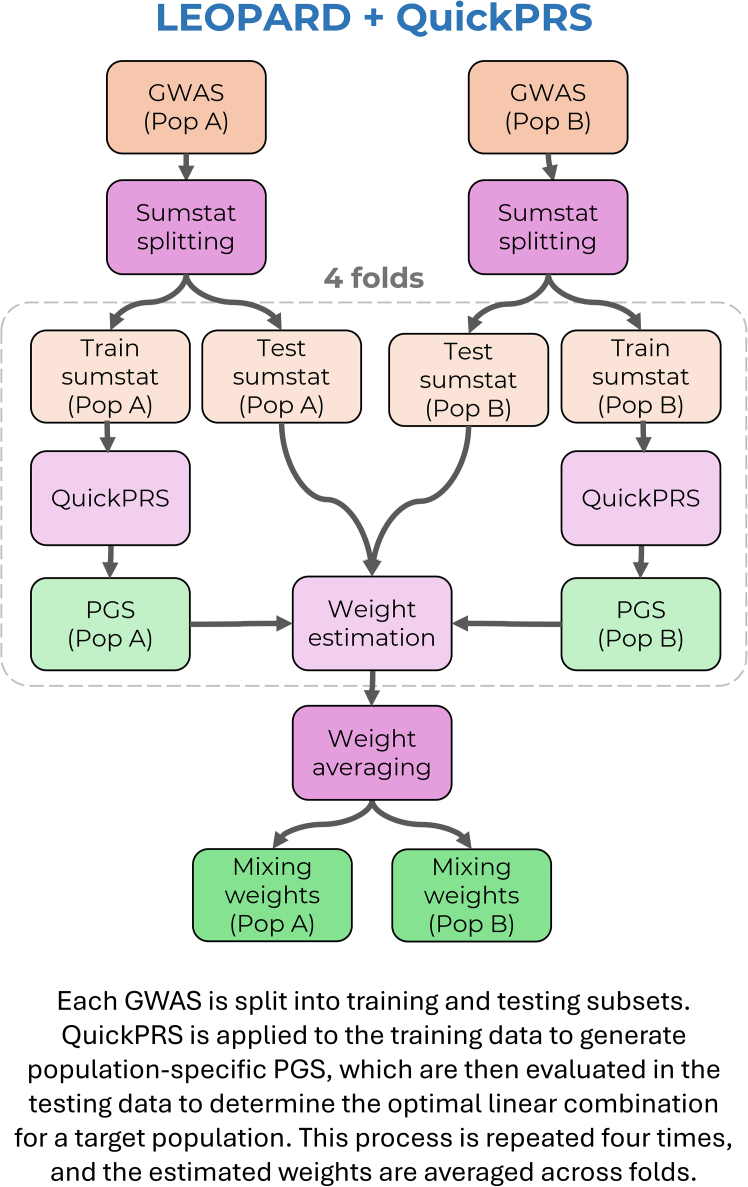
Overview of LEOPARD + QuickPRS approach Pop, population.

This study presents a novel application of X-Wing’s LEOPARD method: LEOPARD is used to estimate the optimal linear combination of population-specific PGSs for independently optimized multi-source approaches. LEOPARD involves three steps: (1) splitting GWASs into training and testing subsets, (2) training the PGS weights in the training subset, and (3) using the testing subset to estimate the appropriate weight of population-specific PGSs for a given target population. Independent linkage disequilibrium (LD) reference data are required for each of these three steps, which is achieved by splitting the 1KG + HGDP reference data into three parts. Given that single-source PGS methods showed broadly similar performance, QuickPRS was used within the LEOPARD analysis to generate the PGS weights (Figure 3). QuickPRS was selected due to the speed and accuracy of its SumStatTune PGS. This assumes that the optimal weights for combining population-specific PGS would not depend strongly on which single-source method was used, and thus QuickPRS would be representative of the others. The PGS weights from LEOPARD were scaled to correspond to each population-specific PGS having an SD of 1, to improve their applicability to scaled PGS from other methods. Note that LEOPARD estimates the optimal linear combination of one PGS from each population. In practice, this means the LEOPARD-derived weights are applied to one PGS per population (specifically, the PGS that was produced by each single-source method’s summary-statistic-only tuning approach).

### Evaluating PGSs

The GWASs used to estimate SNP effect sizes were external to the target sample and held fixed. Within the target dataset, the predictive utility of the PGS was assessed using 10-fold cross-validation. The training subset was used to identify the optimal hyperparameters for each population-specific PGS and to determine the optimal linear combination of these population-specific PGSs. The final PGS model was then evaluated in the held-out test subset. PGS models were evaluated using R, with model evaluation performed using the model_builder_top1.R script within the GenoPred repository (see data and code availability). For multi-source methods, both the tuning of hyperparameters for each population-specific PGS and the determination of mixing weights were performed sequentially using the same training subset within 10-fold cross-validation.

To assess whether the best PGS method varied across traits, an “All” model was also derived. The “All” model considered every PGS method: for single-source approaches it selected the best-performing population-specific PGS, and for multi-source approaches it identified the optimal linear combination of population-specific PGSs. This approach provides a robust benchmark representing the highest achievable performance under both single- and multi-source scenarios, against which other PGS methods can be compared.

### Evaluating prediction accuracy

Prediction accuracy was evaluated as the Pearson correlation between the observed and predicted outcome values. To compare PGS methods and modeling approaches, the correlations between observed and predicted values of each model were statistically compared using the Hotelling-Williams test^40^ as implemented by the “psych” R package’s “paired.r” function, with the correlation between model predictions of each method specified to account for their non-independence. A two-sided test was used when calculating *p* values.

The correlations between predicted and observed values were then pooled across all traits for each PGS method. These correlations (and their variances) were aggregated using the “BHHR” method,^41^ as implemented in the “MAd” R package’s “agg” function. An outcome trait correlation matrix was used in this meta-analytic aggregation to account for the non-independence of the traits within each target population.

### Evaluating linear combination weight estimation

The performance of LEOPARD with QuickPRS in estimating the optimal linear combination of population-specific PGSs was evaluated in three ways. First, the predictive utility of the resulting combined PGS was assessed in the target sample (as described above). Second, the calibration of LEOPARD-derived weights was evaluated by comparing them to observed weights obtained through linear regression of the trait on population-specific PGSs using individual-level data. Calibration was quantified using the root mean squared error (RMSE) between the estimated and observed weights. Third, RMSE was also used to quantify the variability in observed weights when using PGSs derived from different single-source methods, in order to assess whether QuickPRS-based weights are representative for estimating optimal weights across methods. Finally, the performance of LEOPARD for linearly combining population-specific PGS was compared with the “--meta” option in PRS-CSx, which performs inverse-variance meta-analysis of posterior SNP effect sizes. For each method, relative improvement over the individual-level linear combination (IndivTune) was calculated to evaluate whether LEOPARD offers added value over simpler meta-analytic strategies.

### Computational benchmark

GenoPred is a Snakemake pipeline, so the runtime and peak memory of each PGS method were recorded using the Snakemake benchmark functionality.^42^ These benchmarks reflect the time and memory used by each PGS method as implemented in the GenoPred pipeline, and they should not deviate substantially from the resource usage observed in other implementations of the same methods. All analyses were performed using the King’s Computational Research, Engineering and Technology Environment (CREATE).^43^

### Sensitivity analyses

#### Million Veteran Program

To assess the robustness of findings to the choice of African ancestry GWASs, analyses were repeated using publicly available summary statistics from the MVP,^25^ which has substantially larger sample sizes than the UGR GWAS. The same set of 10 traits, GWAS quality control procedures, polygenic scoring methods, tuning strategies, and calibration analyses were applied, with the MVP summary statistics replacing UGR as the African ancestry discovery dataset. Descriptive statistics for the AFR MVP GWAS are provided in Table S2.

#### Downsampled EUR GWAS

To evaluate the impact of GWAS sample size on the performance of polygenic scoring methods, GWAS was performed on downsampled subsets of the UKB EUR sample, with target sample sizes of 5k, 15k, 45k, and 135k per trait. A subset of PGS methods was selected for this analysis based on their performance in the main analysis.

## Results

This study applied 10 PGS methods to GWASs for 10 traits from AFR, EAS, and EUR populations, and evaluated the PGS in independent AFR, EAS, and EUR target samples from the UKB. The predictive utility and computational efficiency of PGS methods were compared when tuned using either individual-level data (IndivTune) or summary statistics alone (SumStatTune) (Figure 2).

### Trait selection and descriptives

Heritability and polygenicity estimates for all 28 available traits, based on GWASs from UGR (AFR), BBJ (EAS), and UKB (EUR) populations, are provided in Table S1. The following 10 traits were selected (covering a range of SNP-based heritability and polygenicity): body mass index, body weight, hemoglobin, HDL-cholesterol, height, mean corpuscular hemoglobin concentration, neutrophil count, platelet count, systolic blood pressure, and total cholesterol. SNP-based heritability estimates (from EUR GWAS) ranged roughly from 5% to 24%, and polygenicity ranged from about 3% to 15%. The median GWAS sample sizes were approximately 13,000 for AFR, 147,000 for EAS, and 297,000 for EUR. The median target sample sizes were approximately 6,000 for AFR, 2,000 for EAS, and 75,000 for EUR. Across traits, the variance explained by the pT + clump PGS (using ancestry-aligned GWAS) ranged from 0.17% to 4.20% in AFR, 0.66%–9.06% in EAS, and 1.88%–17.89% in EUR (Table S3). These results confirm that the study design—comprising the GWAS, target data, and traits selected—had sufficient information to capture polygenic prediction, and that the traits are diverse in genetic architecture.

Analysis of allele frequencies from GWASs conducted in UGR (AFR), BBJ (EAS), and UKB (EUR) indicated broad alignment with their respective reference populations, although fine-scale differences were evident. Principal components 1–5 show strong concordance between each GWAS and its corresponding reference population (Figure S2), though BBJ notably differs from the EAS reference on PC6. Furthermore, fine-scale ancestry composition (Figure S3) reveals that the specific ancestry profiles within each GWAS do not exactly match those of the reference panels.

### Single-source PGS methods

**Figure 4:**
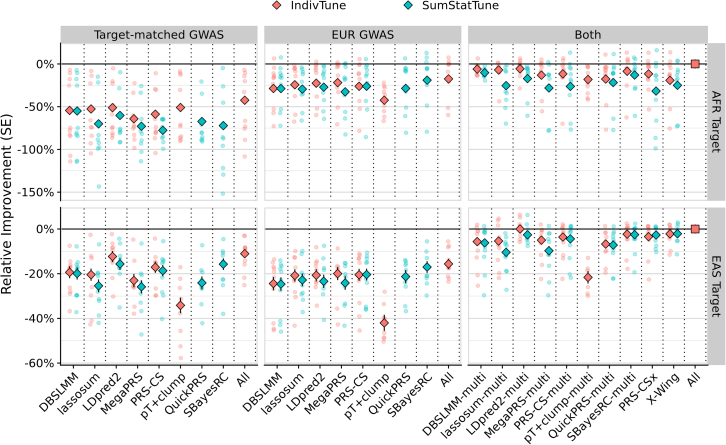
Relative predictive performance of PGS methods in AFR and EAS target populations The *y* axis shows the relative improvement in predictive performance compared with the multi-source “All” model, with error bars representing the standard error. The diamond-shaped points indicate the average difference across traits, with small circular points indicating trait-specific differences. Colors indicate whether PGS methods were trained using individual-level data (IndivTune) or GWAS summary statistics alone (SumStatTune). Facet columns represent the source of the GWAS data used for PGS derivation, including target ancestry-aligned (“Target-matched”) GWAS, European (EUR) GWAS, and combined target ancestry-aligned and EUR GWAS (“Both”). Facet rows show performance in African (AFR) and East Asian (EAS) target populations. The “All” model was derived by selecting the best-performing population-specific PGS across all methods for single-source approaches and identifying the optimal linear combination of population-specific PGSs for multi-source methods. This ensures that the “All” model provides a robust benchmark, reflecting the highest achievable predictive utility under both single- and multi-source scenarios.

The predictive performance of single-source PGSs in AFR and EAS target samples was first evaluated, using either ancestry-aligned or EUR GWASs for training (Figure 4). Across all traits, PGSs trained on EUR GWAS outperformed those trained on AFR GWASs in AFR target samples. This suggests that the larger EUR GWAS sample sizes provided greater predictive power despite ancestry mismatch. In EAS target samples, PGSs trained on EAS GWASs performed similarly to those trained on EUR GWASs. This indicates that the ancestral relevance of the EAS GWAS compensated for its smaller sample size, yielding prediction accuracy comparable to the larger EUR GWAS. As expected, the absolute predictive performance was lower in the AFR target than the EAS target. This likely reflects the smaller sample size of the AFR GWASs and the greater genetic distance between the AFR population and the EUR population (Figure S4).

The relative performance of single-source PGS methods was broadly consistent regardless of which population’s GWAS was used (Figure 4), though two notable exceptions emerged. First, the simple pT + clump method performed relatively well when using AFR GWAS compared with EAS and EUR GWASs—a pattern consistent with sensitivity analyses showing that clumping-based methods tend to perform relatively better when GWAS sample sizes are small (Figure S33). Second, the performance of SumStatTune approaches for MegaPRS, QuickPRS, and SBayesRC performed worse when using AFR GWASs compared with EAS or EUR GWASs.

Models selecting the best PGSs across all methods yielded performance gains (relative to the single best method) of about 17% in AFR targets (*p* = 2 × 10^−3^) and 1% in EAS targets (*p* = 0.41) (Figures 4, S5, and S6; Table S3). This suggests that no single method consistently performs best across all traits with AFR GWASs. In contrast, with EAS GWASs, a single method (LDpred2) performed well across most traits. When using EUR GWASs, SBayesRC performed well across all traits. A model considering PGSs across all methods did not provide a statistically significant improvement over SBayesRC (only +1.8% in AFR target, *p* = 0.25; and +1.7% in EAS target, *p* = 0.44). Detailed trait-specific performance of all PGS methods is provided in Figures S7–S16 and Table S4.

In the EUR target population, the relative performance ranking of the single-source PGS methods was similar to that observed in other populations (Figure S17).

### Multi-source PGS methods

Multi-source PGS methods demonstrated greater predictive power than single-source approaches in both AFR and EAS target populations (Figure 4). Both jointly and independently optimized multi-source methods (which combine multiple ancestry-specific PGSs) generally outperformed the single-source PGSs. For example, LDpred2-multi outperformed the standard single-population LDpred2 PGS by 21.8% in AFR target (*p* = 1 × 10^−28^) and 14.0% in EAS target (*p* = 2 × 10^−9^) (Table S3). The relative improvement of LDpred2-multi over LDpred2 varied across traits, ranging from 0.3% to 37.3% in AFR and 3.0%–22.2% in EAS (Table S4).

On average, the performance was similar across the different multi-source methods**.** LDpred2-multi performed best when individual-level tuning data were available (IndivTune), whereas SBayesRC-multi was the most effective when tuning PGS using summary statistics alone (SumStatTune). Models selecting the best PGS across all multi-source methods yielded an additional improvement of 6% in AFR (*p* = 6 × 10^−4^), but showed no significant improvement in EAS (0%, *p* = 1) (Figure 4).

The jointly optimized methods (PRS-CSx and X-Wing) did not outperform independently optimized approaches. On average, PRS-CSx showed no improvement over its independently optimized counterpart (PRS-CS-multi) in either AFR or EAS targets. X-Wing performed worst with AFR data but with EAS data it achieved results comparable to the best methods. A sensitivity analysis using only 1KG reference individuals (as opposed to 1KG + HGDP) showed no change in performance for any of the methods (Figure S18).

As expected, using TL-PRS to tune an EUR-based PGS with a target-matched GWAS significantly improved prediction accuracy compared with the unadjusted EUR PGS. However, applying TL-PRS to independently optimized multi-source PGSs resulted in only marginal improvements (Figure S19). For example, MTL-SBayesRC-multi—which applies TL-PRS to the SBayesRC-multi scores—improved prediction accuracy by only 1.2% in AFR (*p* = 0.19) and 2.3% in EAS (*p* = 0.02), relative to SBayesRC-multi without TL adjustment.

Multi-source PGS provided a small but statistically significant improvement over using only an EUR PGS (Figures S17, S22, and S23; Table S3). For example, on average, SBayesRC-multi improved prediction accuracy over single-population SBayesRC by 0.1% (*p* = 8 × 10^−4^) when using EUR+AFR data, and by 0.9% (*p* = 5 × 10^−33^) when using EUR+EAS data.

### Impact of tuning PGSs without individual-level data

Tuning PGSs with individual-level data (IndivTune) generally improved prediction accuracy compared with tuning based on GWAS summary statistics alone (SumStatTune). Among single-source methods, the SumStatTune approach performed worst for certain methods when using AFR GWASs. For example, using target-matched GWASs with LDpred2, the IndivTune PGS showed an average relative improvement over the SumStatTune PGS of 22.9% in AFR (*p* = 2 × 10^−10^), 4.0% in EAS (*p* = 6 × 10^−3^), and 2.5% in EUR (*p* = 2 × 10^−87^) (Table S3).

**Figure 5:**
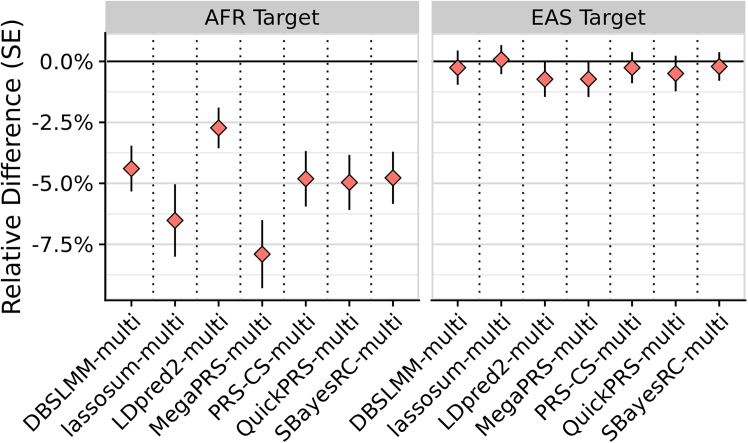
Comparison of prediction accuracy between SumStatTune and IndivTune PGSs using the LEOPARD with QuickPRS framework for combining population-specific scores The *y* axis shows the relative difference in correlation (R) between predicted and observed values, comparing SumStatTune PGS (using only GWAS summary statistics) with IndivTune PGS (using individual-level target data to estimate optimal weights). Error bars represent the standard error of the difference. The *x* axis lists independently optimized multi-source PGS methods, where population-specific PGSs were derived using a summary-statistics-only method from the corresponding single-source method (e.g., LDpred2-auto model). Negative values indicate that SumStatTune performs worse than IndivTune. The left panel shows results for the African (AFR) target population and the right panel for the East Asian (EAS) target population.

**Figure 6:**
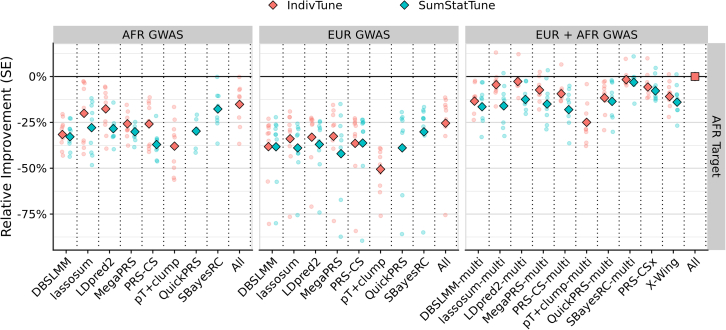
Relative predictive performance of PGS methods in AFR target population using MVP AFR GWAS and UKB EUR GWAS The *y* axis shows the relative improvement in predictive performance compared with the multi-source “All” model, with error bars representing the standard error. The diamond-shaped points indicate the average difference across traits, with small circular points indicating trait-specific differences. The trait “NEU” was excluded as X-Wing failed to complete for this trait. Colors indicate whether methods were trained using individual-level data (IndivTune) or GWAS summary statistics alone (SumStatTune). Facet columns represent the source of the GWAS data used for PGS derivation, including African (AFR) GWAS, European (EUR) GWAS, and combined AFR and EUR GWASs. The “All” model was derived by selecting the best-performing population-specific PGS across all methods for single-source approaches and identifying the optimal linear combination of population-specific PGSs for multi-source methods. This ensures that the “All” model provides a robust benchmark, reflecting the highest achievable predictive utility under both single- and multi-source scenarios.

Among jointly optimized multi-source methods, X-Wing’s summary-statistic tuning approach (LEOPARD) performed well. PRS-CSx also performed well using SumStatTune with EAS data but performed poorly with AFR data. Notably, this study’s novel implementation of LEOPARD with QuickPRS as a SumStatTune approach for independently optimized multi-source methods also showed strong performance. For example, in the case of QuickPRS-multi, tuning the linear combination of population-specific PGS with individual-level data provided a modest relative improvement in AFR (5.2%, *p* = 1 × 10^−5^), but a small improvement in EAS (0.5%, *p* = 0.5) (Figure 5). The poorer performance of LEOPARD in the AFR setting may therefore reflect the smaller sample size of the UGR GWAS. Sensitivity analyses support this interpretation: LEOPARD’s performance improved with increasing GWAS sample size in the downsampled EUR analyses (Figure S32) and also improved when using the larger AFR GWAS from the MVP (Figure 6). In the latter case, when using MVP AFR GWAS with QuickPRS-multi, the IndivTune PGS outperformed the SumStatTune PGS by only 2.3% on average (*p* = 2 × 10^−4^) (Figure S29; Table S5), highlighting the narrowing performance gap as GWAS size increases.

To assess how well LEOPARD-estimated weights approximated those obtained with individual-level data, root mean squared error (RMSE) was used to quantify calibration. RMSE was used to compare the observed weights across different methods; weights derived from QuickPRS showed a low RMSE across methods (Figures S22–S24), suggesting high concordance across methods and supporting the use of QuickPRS with LEOPARD for computationally efficient weight estimation. Notably, the RMSE of LEOPARD-estimated weights, as well as the concordance of observed weights across methods, was lower when using the AFR GWAS compared with the EAS GWAS.

LEOPARD and inverse-variance meta-analysis (as implemented in PRS-CSx) showed broadly similar performance when combining population-specific PGS (Figures S30 and S31). However, LEOPARD yielded a notable improvement in relative prediction accuracy for a subset of traits where there was a large imbalance in GWAS sample sizes—particularly when using smaller discovery GWAS from the UGR—suggesting that it may provide more robust performance than inverse-variance weighting in settings with highly unbalanced data.

Currently, there is no summary-statistic-based approach for tuning TL-PRS. This means TL-PRS adjustments only can be applied when individual-level data are available.

### Computational benchmark

The average time and memory required by each method are shown in Table 1. With 10 CPU (central processing unit) cores available and using the recommended tuning approach for each method, most methods complete within 30 min. However, some methods were substantially slower: PRS-CS, PRS-CSx, and X-Wing required roughly 4.4, 6.8, and 34.1 h per GWAS, respectively. All methods were run using the same 1,204,449 HapMap3 variants overlapping with the 1KG + HGDP reference, ensuring comparability in computational demands. Computational resource requirements are expected to increase with the number of variants considered, underscoring the importance of computational efficiency. In contrast, resource use is largely independent of GWAS sample size, as summary statistics (rather than individual-level data) are used.

**Table 1:** Computation resource required for different methods as implemented within GenoPred, using 10 cores, including both IndivTune and SumStatTune approaches where available

Method	Time (h)	Memory (GB)
DBSLMM	0.15	1.15
lassosum	0.08	7.30
LDpred2	0.38	20.54
MegaPRS	0.872	11.92
PRS-CS	4.40^b^	10.57
pT + clump	0.02	0.83
QuickPRS	0.06	4.41
SBayesRC	0.38	4.4
PRS-CSx^a^	6.84^b^	15.18
X-Wing^a^	34.12^c^	48.7
LEOPARD (QuickPRS)^a^	0.23	8.03

a Time per GWAS (total time divided by 2). Memory with two of input GWASs.

b Time taken with grid search (4 phi parameter + auto). Auto model only will be 20% of total.

c Time taken with X-Wing with LEOPARD. Without LEOPARD, will be 20% of total.

Runtime depends strongly on the tuning procedure used. The reported times for PRS-CS and PRS-CSx assume a grid search over five different global shrinkage parameters (phi). If instead the SumStatTune (auto) approach is used, their runtimes drop to roughly 20% of those defaults—only about 0.88 and 1.37 h, respectively. Similarly, X-Wing runs much faster if LEOPARD (the SumStatTune weighting step for population-specific PGS) is skipped, cutting its runtime to ∼6.82 h (about 20% of the default). This speed-up occurs because the LEOPARD step requires re-estimating SNP effects four additional times per GWAS. For the remaining PGS methods, runtime is not expected to vary substantially between the IndivTune and SumStatTune approaches.

X-Wing, an extension of PRS-CS and PRS-CSx, is inherently slower than other PGS methods. In contrast, this study’s novel application of LEOPARD with QuickPRS enables much faster estimation of population-specific PGS weights—completing in just 14 min per GWAS.

Thus, the independently optimized multi-source approach—using single-source PGS methods and combining them with LEOPARD+QuickPRS weights—is far more computationally efficient than jointly optimized PGS methods like PRS-CSx or X-Wing. For instance, with 10 cores and individual-level tuning, applying LDpred2 to two GWASs takes 46 min, whereas X-Wing takes 13.64 h. If using a summary-statistic-only tuning approach, applying SBayesRC to two GWASs and estimating the linear combination using LEOPARD+QuickPRS takes 1.25 h, compared with 68.24 h for X-Wing.

With 10 cores, adjusting a single PGS model using TL-PRS took approximately 20 min and required 31 GB of memory.

### Sensitivity analyses

To evaluate the robustness of our findings, sensitivity analyses were conducted using (1) alternative AFR GWAS summary statistics from the Million Veteran Program (MVP), and (2) downsampled EUR GWASs to assess the impact of discovery sample size. Results were broadly consistent with the main findings. When using the MVP AFR GWAS, SBayesRC and SBayesRC-multi showed the highest prediction accuracy, regardless of whether IndivTune or SumStatTune was applied (Figure 6). LDpred2 also performed comparably to SBayesRC when using IndivTune. These findings are consistent with the main analysis despite ancestry composition analysis indicating that the MVP AFR GWAS included approximately 15% European ancestry (Figures S2 and S3). Analyses using MVP AFR GWAS and downsampled EUR GWAS also confirmed that LEOPARD performed better with larger GWAS sample sizes (Figures S29 and S32), and that clumping-based methods performed relatively better at smaller sample sizes (Figure S33). Together, these results underscore the influence of both GWAS sample size and ancestry composition on PGS performance and demonstrate the robustness of the main findings. Further details of the sensitivity analysis results are provided in the supplemental information.

## Discussion

This study presented a comprehensive comparison of leading PGS methods applied to datasets from multiple ancestral populations. It evaluated predictive performance and computational efficiency of both single-source and multi-source PGS methods, within and across populations, using individual-level tuning and summary-statistic-only tuning approaches. As expected, multi-source methods offered significantly improved prediction over single-source methods. Notably, independently optimized multi-source methods achieved competitive prediction accuracy while being substantially more computationally efficient than the jointly optimized multi-source methods. Additionally, this study developed a novel extension of the LEOPARD method using QuickPRS to provide a summary-statistic-based approach for weighting population-specific PGS from independently optimized multi-source methods.

All the methods evaluated in this study have been integrated into the GenoPred pipeline, which is publicly available and designed for broad accessibility (see data and code availability). These findings, alongside prior research, underscore the importance of incorporating GWAS data from multiple populations to optimize PGS prediction in ancestrally diverse samples. Importantly, computationally efficient, summary-statistic-only methods (now fully implemented in GenoPred) offer practical solutions for researchers working with limited individual-level data or resources.

This study can guide researchers in selecting appropriate PGS methods based on the availability of GWAS data, target data, and computational resources. For large EUR GWASs, SBayesRC demonstrated superior performance across all traits—notably, without needing any individual-level tuning data. SBayesRC also performed well with the large EAS BBJ GWAS and the AFR MVP GWAS, performing comparably to LDpred2 when individual-level tuning data were available. In contrast, the smaller UGR AFR GWAS showed a more pronounced advantage for LDpred2, where several individual-level tuned methods performed similarly. This suggests that LDpred2’s relative advantage in the UGR analysis may have been driven by the small GWAS sample size rather than by method superiority in AFR populations. In general, no single method consistently outperformed others across traits with AFR UGR GWAS, indicating that tuning across methods may enhance prediction accuracy in low-powered GWAS settings.

In practice, for AFR or EAS GWASs, when individual-level training data are available, to maximize prediction accuracy one should apply both SBayesRC and LDpred2 and select the best-performing PGS across methods. If individual-level training data are unavailable or a simpler workflow is desired, SBayesRC alone is a strong choice. When GWAS data from multiple populations are accessible, population-specific PGS can be linearly combined using either individual-level data or the LEOPARD method (which requires summary statistics only). Given the high similarity between population-specific weights across PGS methods, a computationally efficient method like QuickPRS is suitable for generating the LEOPARD weights. This strategy (implemented in the GenoPred pipeline) greatly improves efficiency without sacrificing accuracy.

This study indicates that currently available jointly optimized multi-source methods do not offer an advantage over independently optimized multi-source methods, and they come with a much higher computational cost. This conclusion contrasts with two previous benchmarking studies reporting that jointly optimized methods were the best approach.^18^^,^^44^ However, those studies were limited to a narrower set of methods and did not evaluate direct combinations of population-specific PGSs using independently optimized models. By incorporating a broader range of methods—including those that linearly combine ancestry-specific scores—and evaluating multiple tuning strategies, this study reveals that simpler approaches typically exceed the performance of jointly optimized models. These findings are further supported by the recent SBayesRC study,^19^ which suggests other methodological features, such as incorporating functional annotations, provide a more significant benefit than joint optimization alone. It is possible that future jointly optimized multi-source methods, especially if they incorporate such features, could surpass independently optimized approaches. For now, though, independently optimized approaches remain the recommended choice given their computational efficiency.

Although the TL-PRS post hoc adjustment improved prediction when applied to a single-source PGS (compared with no adjustment), it showed no meaningful improvement when applied to jointly optimized multi-source PGSs. Within the scenarios tested in this study, the additional computational burden and tuning complexity of TL-PRS did not justify its use for multi-source PGSs.

Several limitations of this study should be acknowledged. First, the reference populations were defined broadly and had limited sample sizes, which may have impacted PGS performance due to LD reference panel misspecification. Although this study did not directly evaluate performance under varying degrees of LD mismatch, the top-performing PGS methods were consistent across GWASs with differing degrees of ancestry alignment to the LD reference panel. Since in-sample LD data are rarely available, it is important that PGS methods remain robust when using publicly available LD reference datasets.

Second, using HapMap3 variants as the default in GenoPred might influence the relative performance of PGS methods. While denser genome coverage could enhance methods that leverage functional annotations and fine-scale LD structure, such as MegaPRS, QuickPRS, and SBayesRC,^19^ applying most PGS methods to dense SNP sets is not currently computationally feasible. Moreover, using a denser variant set increases the likelihood of poor SNP overlap with external GWAS or target datasets, potentially reducing the generalizability of the resulting PGS models. Future development of GenoPred will aim to support denser SNP sets, though this lies beyond the scope of the present study.

Third, this study did not evaluate approaches for admixed target individuals. Future research could investigate scoring approaches that account for local ancestry when aggregating population-specific PGSs for an individual.^45^^,^^46^

Finally, the scenario where a single GWAS includes individuals from multiple ancestries was not explicitly addressed. In such cases, it is recommended to either specify a reference population that matches the majority ancestry or to generate a custom reference dataset that reflects the ancestry proportions present in the GWAS.

In conclusion, as GWAS data from diverse populations become increasingly available, multi-source approaches should be prioritized to enhance prediction accuracy in ancestrally diverse target samples. The GenoPred pipeline facilitates this by providing an accessible, robust, and computationally efficient framework to apply state-of-the-art PGS methods, even in the absence of individual-level tuning data. Future methodological work should aim to integrate the strengths of single-source methods into new jointly optimized multi-source methods to maximize predictive performance. Nevertheless, despite the gains from multi-source methods, large disparities in predictive accuracy between European and non-European target populations remain. Closing this gap will require significantly expanding and improving the diversity of GWAS datasets, which is crucial for more equitable and accurate polygenic prediction moving forward.

## Data and code availability

GenoPred homepage: https://opain.github.io/GenoPred/.

A complete summary of the code used to produce the results of this study is available on the GenoPred website: https://opain.github.io/GenoPred/CrossPop.html.

snp_ancestry_summary bigsnpr tutorial: https://privefl.github.io/bigsnpr/articles/ancestry.html.

GreedyRelated: https://gitlab.com/choishingwan/GreedyRelated.

QuickPRS: https://dougspeed.com/quick-prs/.

model_builder_top1.R: https://github.com/opain/GenoPred/blob/gwas_grouping/Scripts/model_builder/model_builder_top1.R.

GWAS summary statistics from BBJ, UGR, and MVP were publicly available (see Tables S1 and S2). The UK Biobank data was accessed via project 82087. For access, go to https://www.ukbiobank.ac.uk/enable-your-research/apply-for-access. HapMap3 SNP-list: https://doi.org/10.5281/zenodo.7773502.

## Acknowledgments

O.P. thanks Michelle Kamp and Florian Privé for feedback on the manuscript; Doug Speed, Eva Krapohl, and Remo Monti for insightful discussions; Ammar Al-Chalabi for fellowship support; and Cathryn Lewis for contributions to the broader GenoPred research program. O.P. also thanks the developers of all PGS methodologies evaluated in this study.

O.P. is supported by a Sir Henry Wellcome Postdoctoral Fellowship (222811/Z/21/Z). The funders had no role in study design, data collection and analysis, decision to publish, or preparation of the manuscript.

This research was conducted under UK Biobank application 82087.

## Declaration of interests

O.P. provides consultancy services for UCB Pharma.

## Declaration of generative AI and AI-assisted technologies in the writing process

During the preparation of this work the author used OpenAI’s ChatGPT in order to improve the clarity and grammar of the manuscript. After using this tool, the author reviewed and edited the content as needed and takes full responsibility for the content of the publication.

## Supplemental information

Data S1. Tables S1–S5

Document S2. Article plus supplemental information
