# Supplementary material for: Leveraging global genetics resources to enhance polygenic prediction across ancestrally diverse populations
Source: HGG Adv. 2025 Jul 18;6(4):100482. doi: 10.1016/j.xhgg.2025.100482 (PMC12536657; doi:10.1016/j.xhgg.2025.100482)
Supplement: Document S1. Figures S1–S33 [file mmc1.pdf]

HGGA, Volume *6*

Supplemental information

Leveraging global genetics resources
to enhance polygenic prediction
across ancestrally diverse populations

Oliver Pain

Supplementary Information for “Leveraging Global Genetics Resources to Enhance Polygenic Prediction Across Ancestrally Diverse Populations”

Oliver Pain[1]

1 = Maurice Wohl Clinical Neuroscience Institute, Department of Basic and Clinical Neuroscience, Institute of Psychiatry, Psychology and Neuroscience, King’s College London, London, United Kingdom.

Corresponding author: Oliver Pain (oliver.pain@kcl.ac.uk)

Sensitivity analysis: Million Veteran Program

To evaluate the robustness of our findings, analyses were repeated using AFR ancestry GWAS summary statistics from the Million Veteran Program (MVP), which had a median sample size of 114,918 individuals. The overall pattern of results remained consistent with the main analyses.

Among single-source methods using the MVP AFR GWAS alone, SBayesRC delivered the highest prediction accuracy on average, despite not requiring individual-level tuning data. LDpred2 also performed well when individual-level tuning data were available. In contrast, pT+clump performed relatively poorly compared to its performance when using the UGR GWAS. This finding suggests that larger GWAS sample sizes can influence the effectiveness of clumping-based methods, congruent with the downsampling sensitivity analysis (described below).

When combining the AFR MVP GWAS with the EUR UKB GWAS, SBayesRC-multi was the best-performing multi-source method, regardless of whether tuning used individual-level data or summary statistics alone. PRS-CSx showed improved performance over PRS-CS-multi, with an average relative improvement of 4% ($p = 1\times10^{-4}$) with IndivTune. However, PRS-CSx was outperformed by several independently optimised multi-source methods. For example, with IndivTune, on average SBayesRC-multi showed a relative improvement of 4.3% ($p = 3\times10^{-3}$) over PRS-CSx. These findings reinforce conclusions from the main analysis.

There was little added benefit from considering all methods ("all-model"), likely because the top-performing methods already captured most of the predictive signal. For single source methods applied to AFR GWAS, the all model did not provide a statistically significant improvement over the best single method (relative improvement 3%, $p = 0.06$). For multi-source methods, the all mode also did not provide a statistically significant improvement over the single best method (relative improvement 1.7%, $p = 0.18$).

LEOPARD with QuickPRS performed well as a SumStatTune approach for independently optimised multisource methods. For QuickPRS-multi, IndivTune PGS only provided an average relative improvement of 2.3% ($p = 2\times10^{-4}$) over the SumStatTune PGS. This performance is better than when using the UGR sumstats indicating LEOPARD performs better with larger sample sizes, again congruent with the downsampling sensitivity analysis (described below). When combining MVP and UKB GWAS, LEOPARD and the PRS-CSx --meta approach showed similar performance for weighting population-specific PGS.

Finally, allele frequency analysis revealed that the MVP AFR GWAS reflect ~15% European ancestry, while the UKB (EUR), BBJ (EAS), and UGR (AFR) GWAS were >99% ancestry-homogeneous (Figures S2–S3). These findings further support the robustness of our conclusions across GWAS sources, sample sizes, and in the presence of modest admixture.

Sensitivity analysis: Downsampled EUR GWAS

PGS methods were evaluated using downsampled EUR UKB GWAS to assess the impact of GWAS sample size on performance. For single-source methods, the sensitivity analysis focused on those included in the main analysis and those that showed variable performance across AFR, EAS, and EUR GWAS — specifically DBSLMM, LDpred2, pT+clump, QuickPRS, and SBayesRC. Most single-source methods and tuning approaches maintained consistent relative performance across GWAS sample sizes, with the exception of pT+clump, which performed relatively well at smaller sample sizes (Figure S33).

The performance of LEOPARD with QuickPRS (i.e., SumStatTune PGS) was also evaluated across a range of EUR GWAS sample sizes, paired with the EAS BBJ GWAS. Results showed that as GWAS sample size increased, the relative performance of SumStatTune PGS improved compared to IndivTune PGS. Specifically, SumStatTune QuickPRS-multi PGS were 3.2%, 2.9%, 1.9%, and 0.5% worse than IndivTune PGS when UKB GWAS sample sizes were 5k, 15k, 45k, and 135k, respectively (Figure S32). Consistent with this, the LEOPARD-estimated linear combination weights were more accurately calibrated to the observed weights as GWAS sample size increased, with RMSE values of 0.21, 0.15, 0.13, and 0.10 at the corresponding GWAS sizes.

These sensitivity analyses show that LEOPARD's performance improves as GWAS sample size increases. However, its performance using the MVP AFR GWAS is lower than expected based on the downsampled EUR GWAS results. This suggests that, beyond GWAS sample size, other factors may influence LEOPARD's performance. Two likely contributors are LD misspecification between the reference and GWAS populations, and imperfect genetic correlation between the GWAS and target samples. While further research is needed to quantify the impact of these factors, our results demonstrate that LEOPARD performs well under the realistic cross-population conditions evaluated in this study.

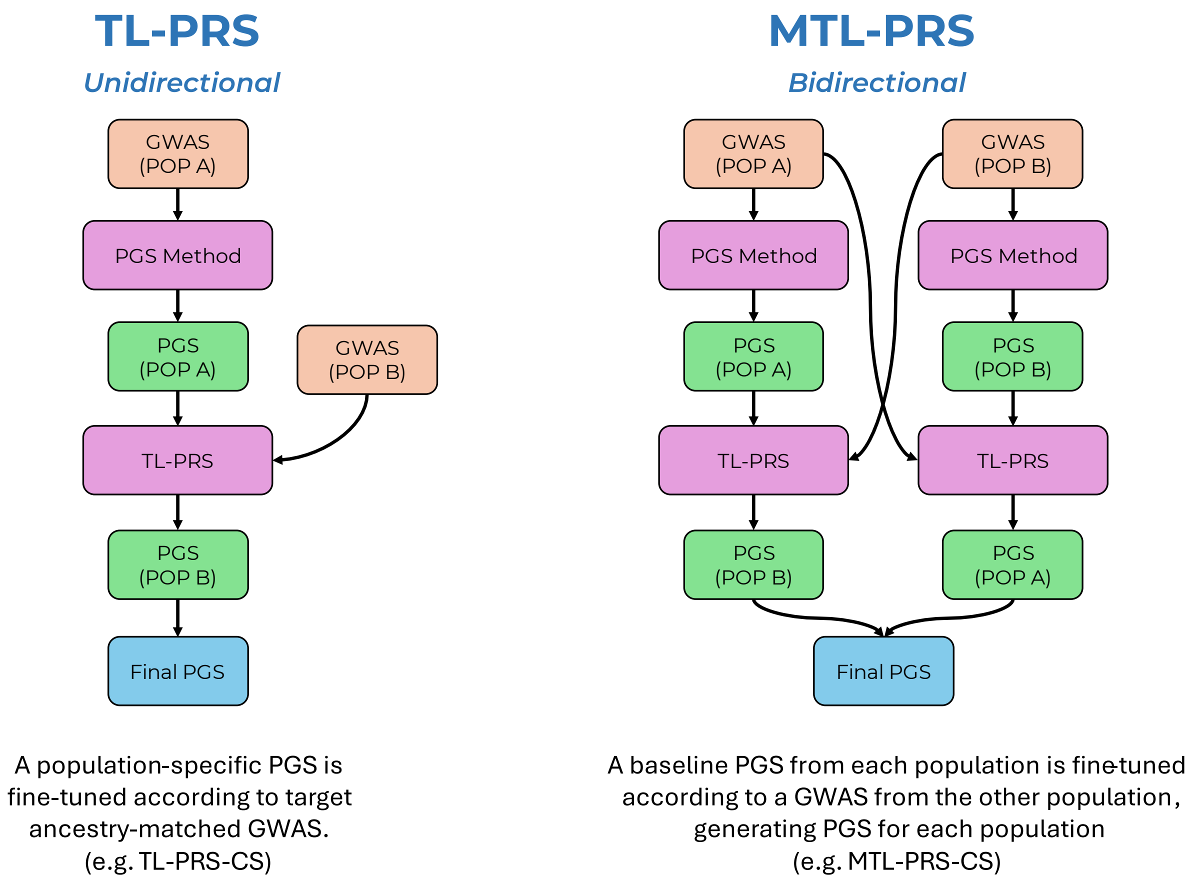

Figure S1. Schematic representation of TL-PRS method. TL-PRS involves tuning an existing PGS model from population A based on GWAS summary statistics from population B. An extension is MTL-PRS, whereby TL-PRS is used bidirectionally to tune PGS models from population A and B using GWAS summary statistics from population B and A respectively, generating two population-specific PGS that can then be linearly combined.

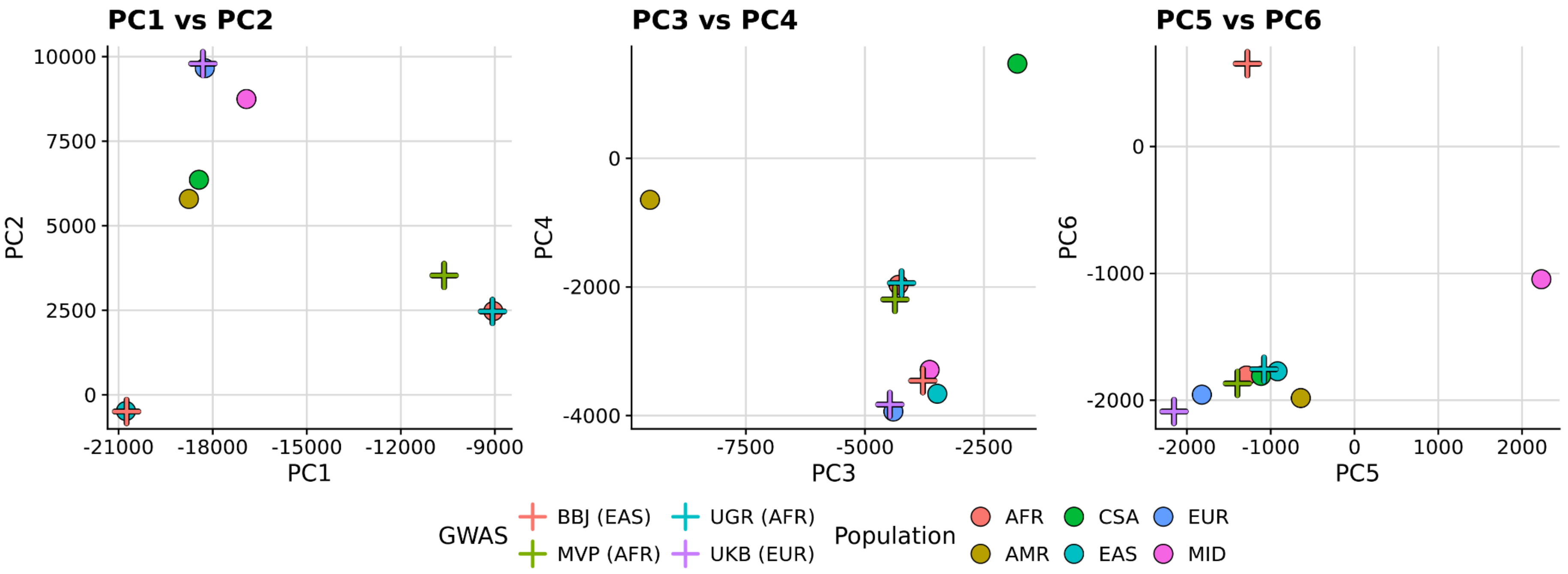

Figure S2. Principal component analysis of GWAS of BMI and reference populations based on allele frequencies. Principal components 1 to 6 (PC1-6) derived from the 1KG+HGDP reference panel were projected into each BMI GWAS dataset based on allele frequency. Circles represent reference populations, and crosses indicate the projected coordinates of each GWAS dataset.

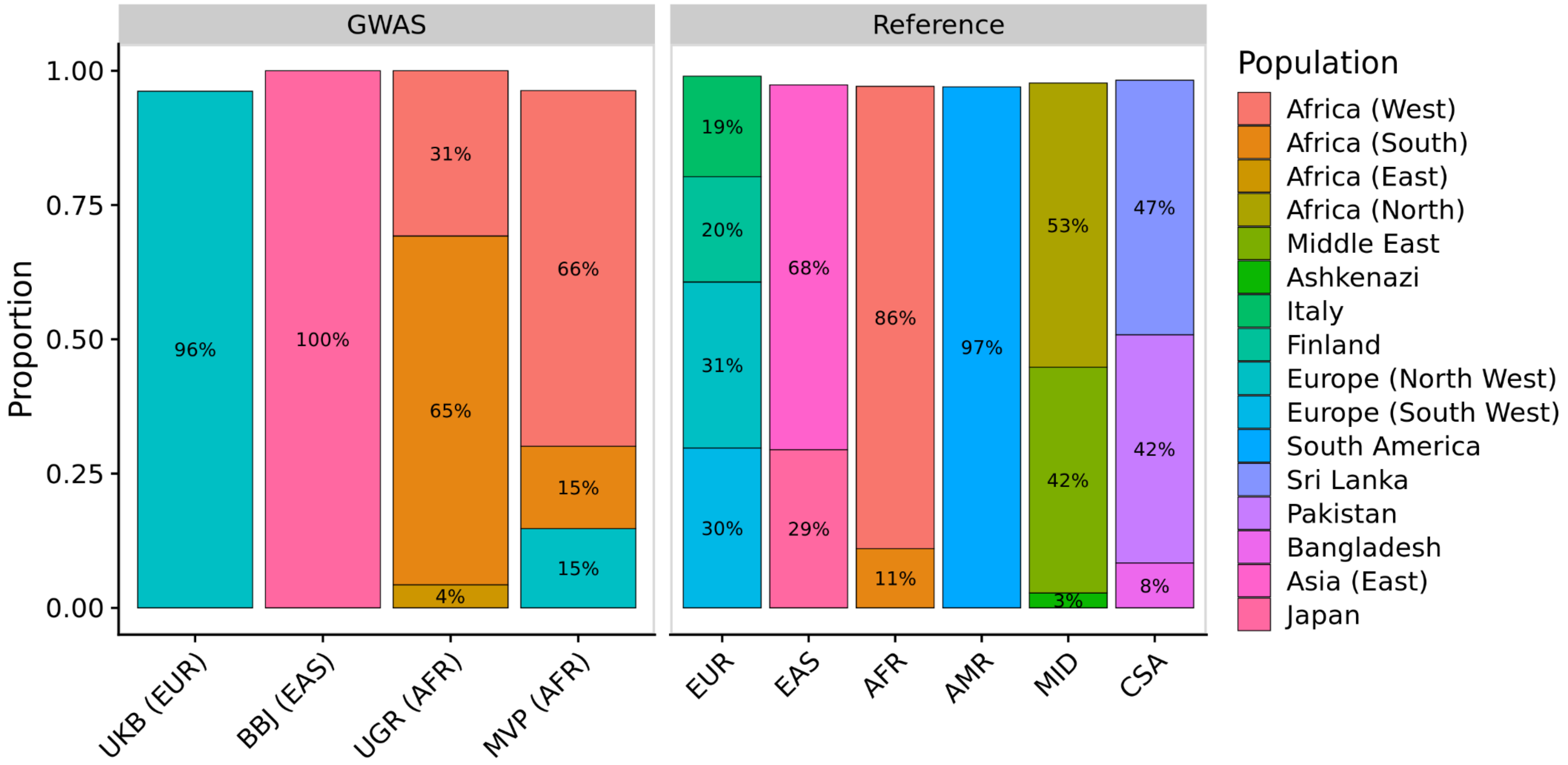

Figure S3. Estimated ancestry composition of BMI GWAS and 1KG+HGDP reference populations based on allele frequencies. Ancestry proportions were estimated using the snp_ancestry_summary function from the bigsnpr R package. The left panel shows the ancestry composition of the BMI GWAS samples used in this study. The right panel shows the estimated ancestry composition of the 1KG+HGDP reference superpopulations. Only population groups contributing more than 2% to a given GWAS or reference sample are displayed. Labels within each bar indicate rounded percentage contributions.

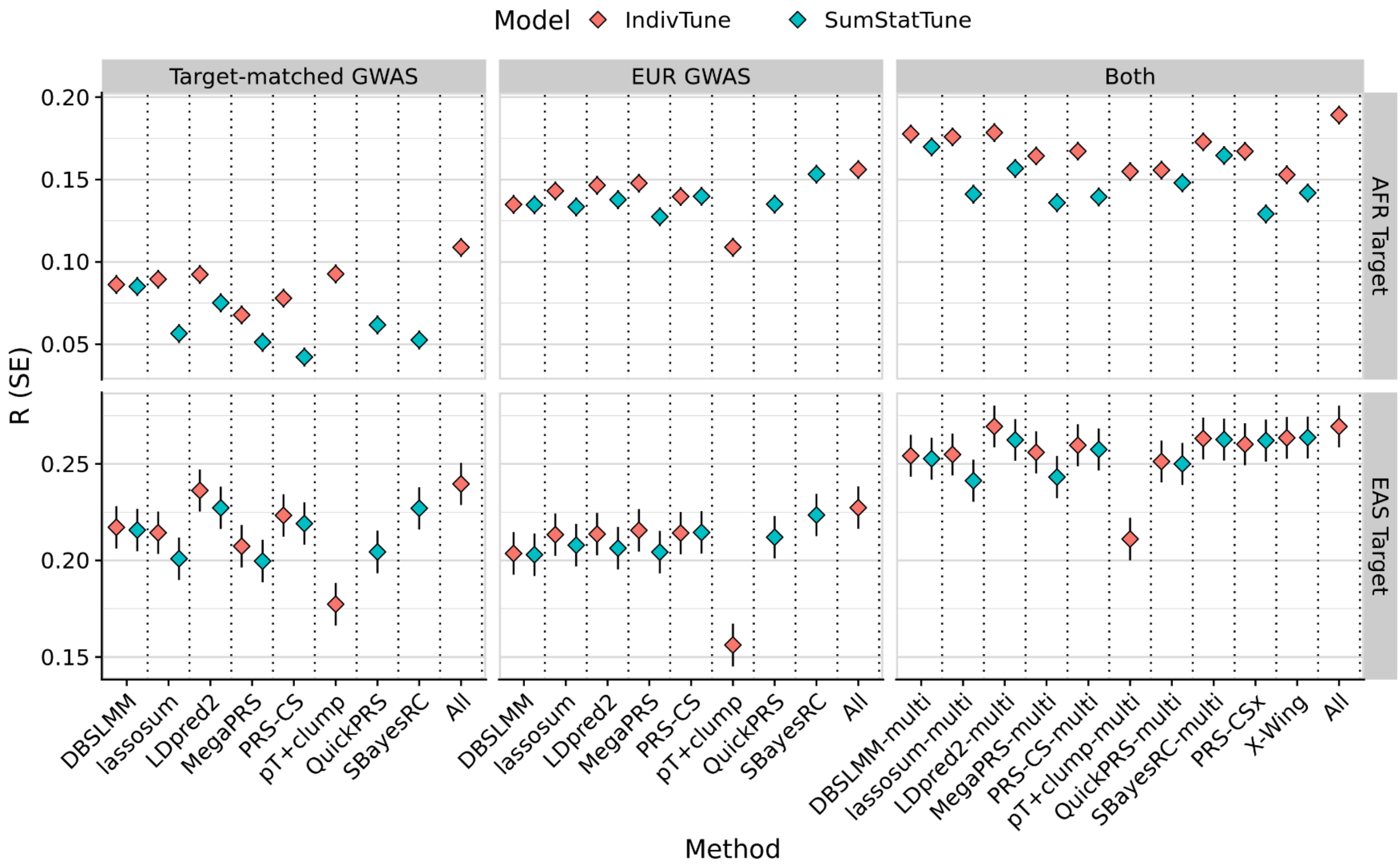

Figure S4. Average absolute predictive utility of PGS methods in AFR and EAS target populations. The y-axis indicates the average correlation between predicted and observed values across traits, with error bars showing the standard error. Colours distinguish whether PGS methods were trained using individual-level data (IndivTune) or GWAS summary statistics alone (SumStatTune). 'Target-matched GWAS', 'EUR GWAS', and 'Both' facets show PGS performance using target ancestry-aligned, European, or combined GWAS data, respectively. 'AFR Target' and 'EAS Target' facets show performance in AFR and EAS samples. 'All' models in the 'Both' facet represent the best population-specific PGS selected across multi-source methods. In the 'Target-matched GWAS' and 'EUR GWAS' facets, 'All' models represent the best population-specific PGS from single-source methods.

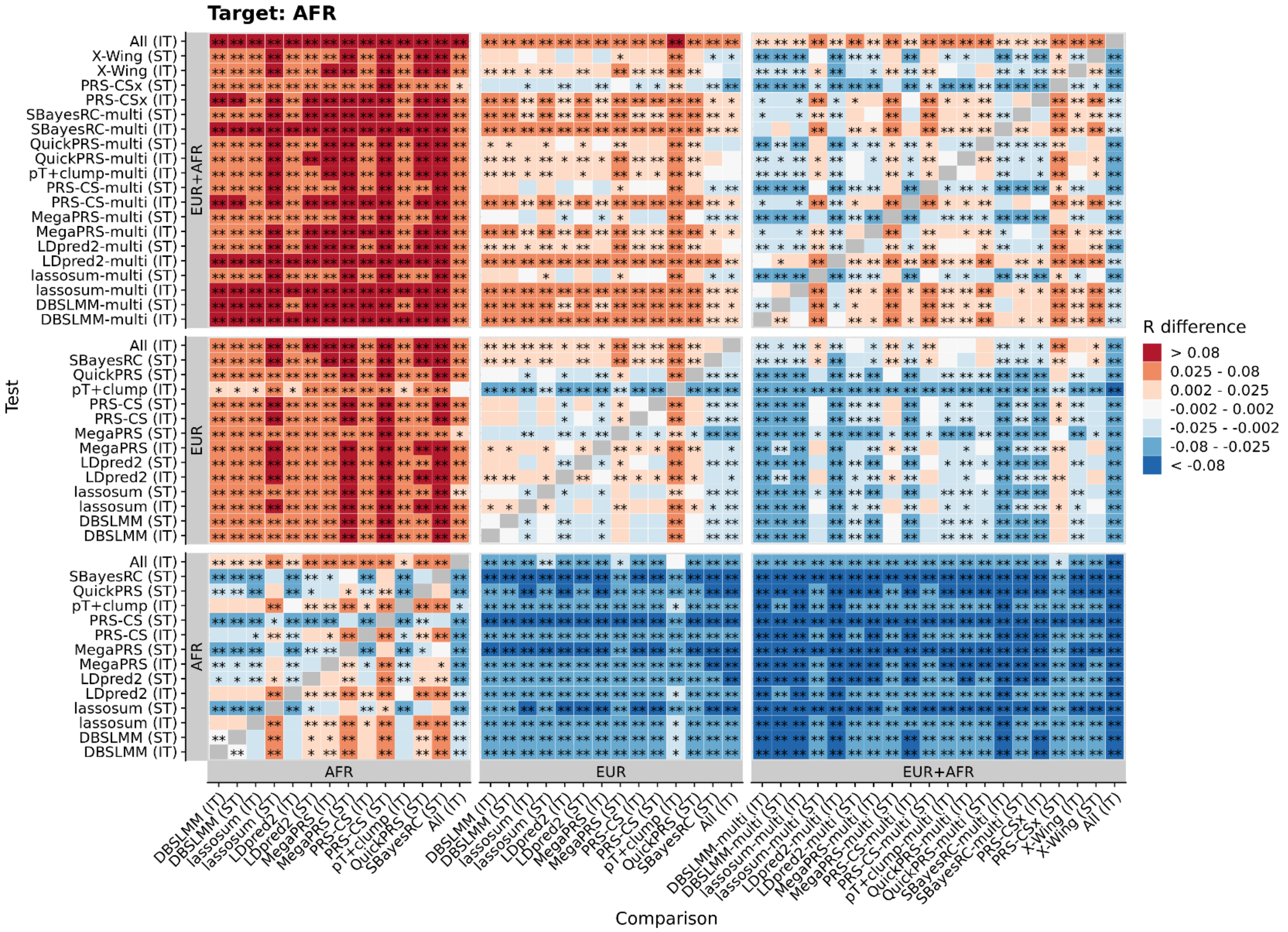

*Figure S5. Pairwise comparison between all methods in AFR target sample, showing average difference in observed-expected correlation. R difference = Test correlation minus Comparison correlation. Red/orange colouring indicates the Test method (shown on Y axis) performed better than the Comparison method (shown on X axis). Shows only results based on the UKB target sample when using the 1KG reference. * = $p<0.05$ * = $p<1\times10^{-3}$. P-values are two-sided. IT = IndivTune, PGS model tuned using individual-level data. ST = SumStatTune, PGS model tuned using GWAS summary statistics alone.*

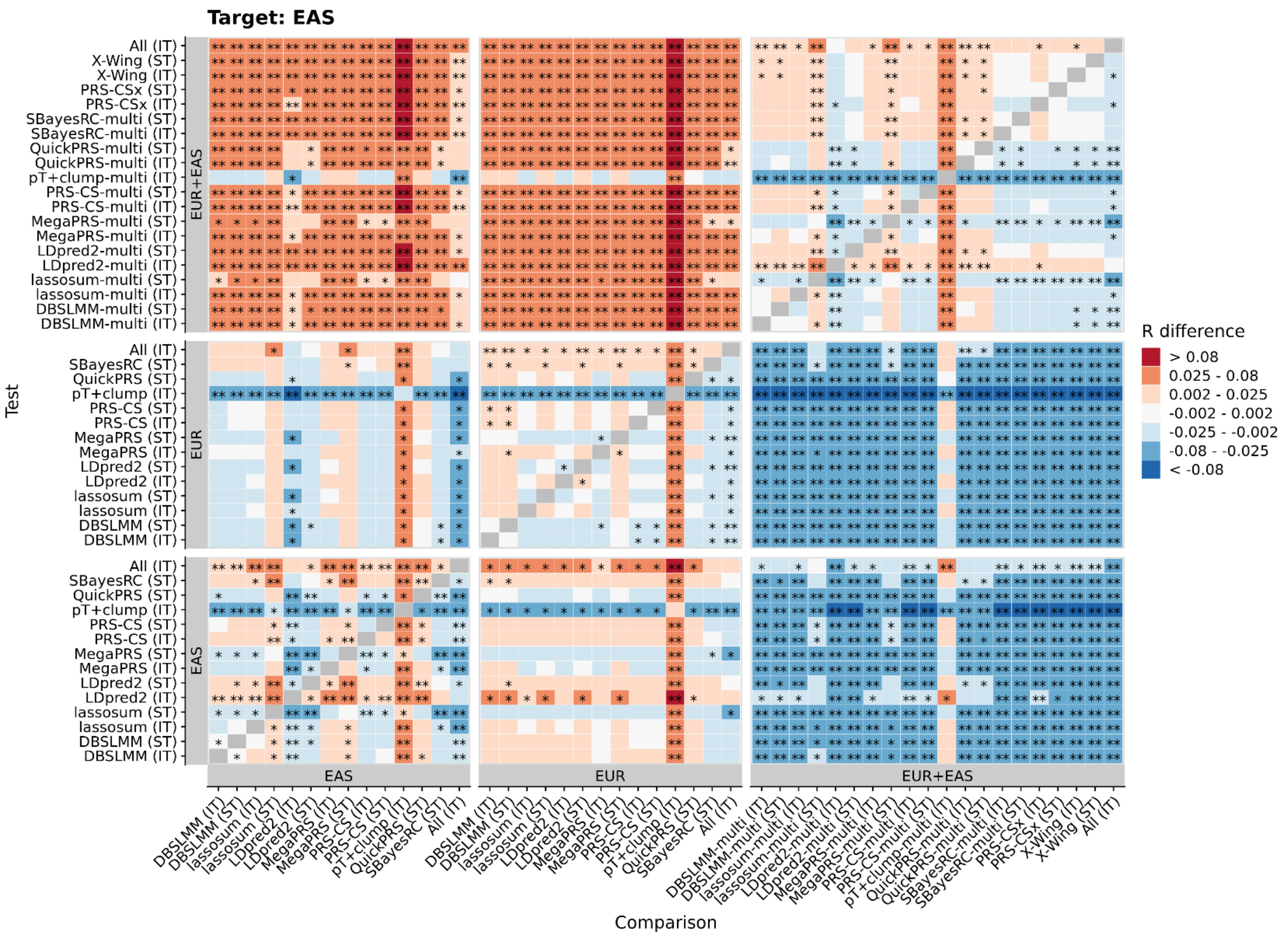

*Figure S6. Pairwise comparison between all methods in EAS target sample, showing average difference in observed-expected correlation. R difference = Test correlation minus Comparison correlation. Red/orange colouring indicates the Test method (shown on Y axis) performed better than the Comparison method (shown on X axis). Shows only results based on the UKB target sample when using the 1KG reference. * = p<0.05 * = p<1×10−3. P-values are two-sided. IT = IndivTune, PGS model tuned using individual-level data. ST = SumStatTune, PGS model tuned using GWAS summary statistics alone.*

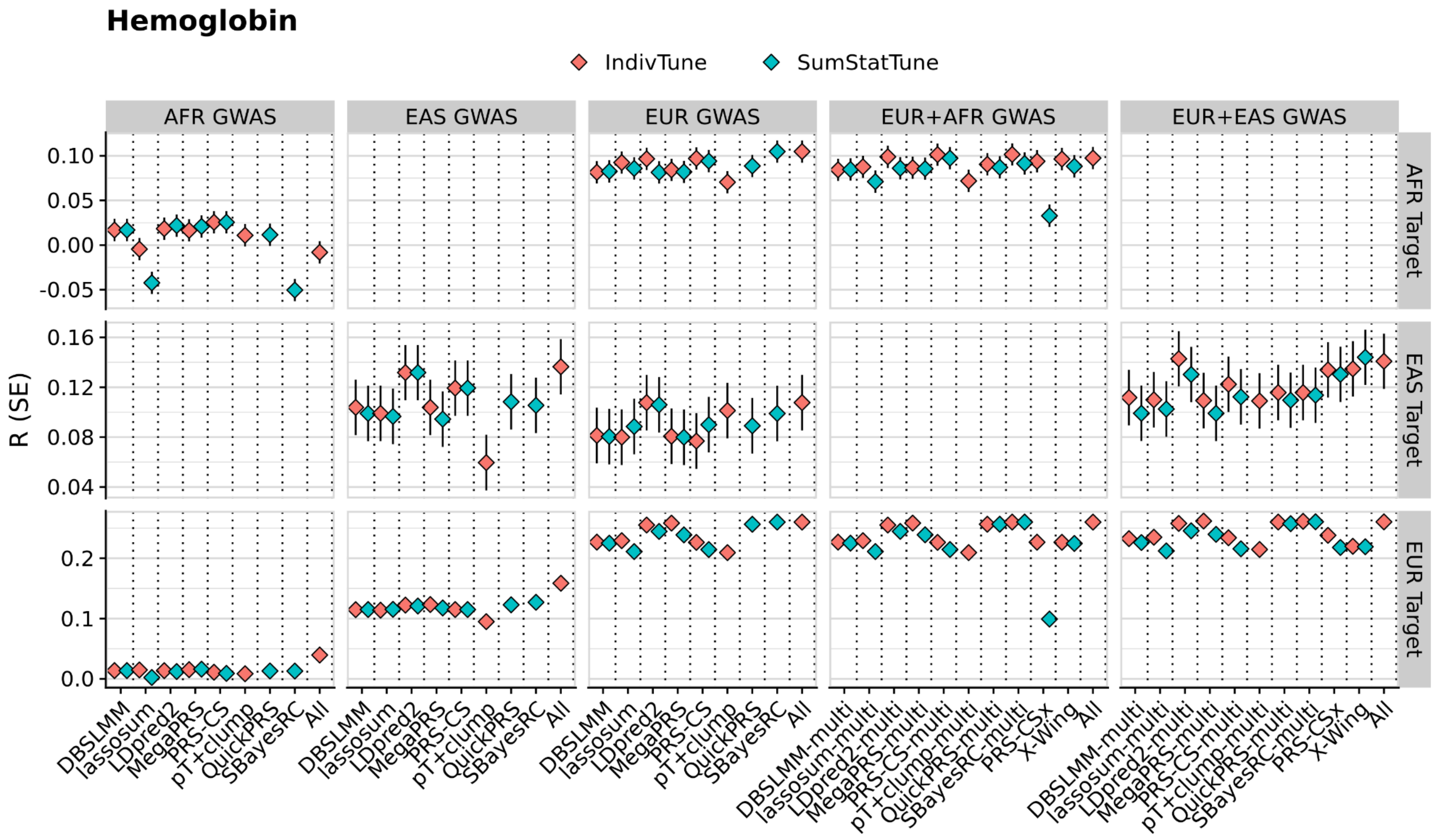

Figure S7. Predictive utility of PGS methods for Hemoglobin. The y-axis shows the correlation (R) between predicted and observed trait levels, with error bars representing the standard error. Colours differentiate between PGS methods trained using individual-level data (IndivTune) and those trained using GWAS summary statistics (SumStatTune). Facet columns indicate the GWAS data source used for PGS derivation, including African (AFR GWAS), East Asian (EAS GWAS), European (EUR GWAS), combined African and European (EUR+AFR GWAS), and combined East Asian and European (EUR+EAS GWAS) data. Facet rows represent performance in African (AFR Target), East Asian (EAS Target), and European (EUR Target) populations.

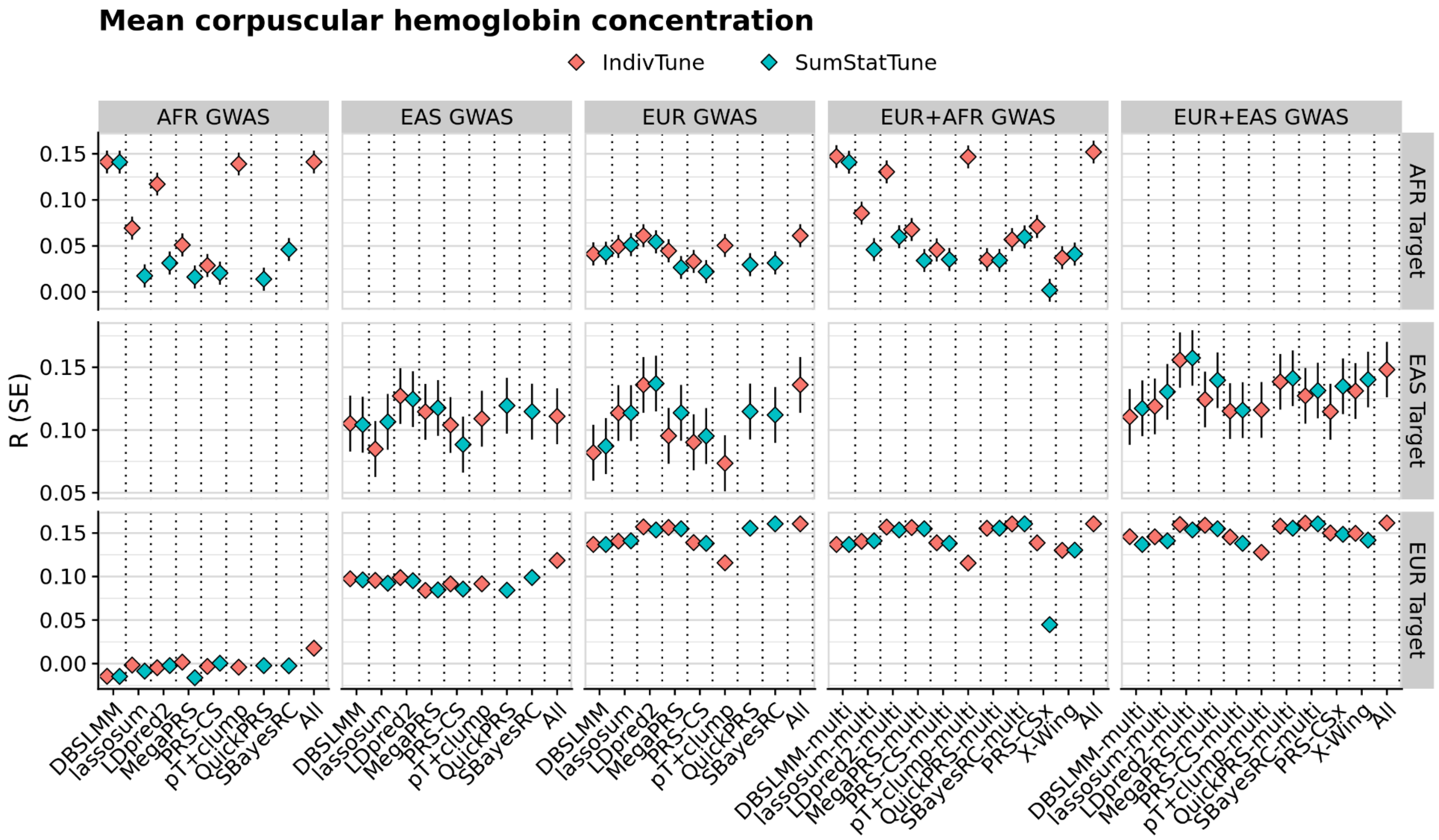

Figure S8. Predictive utility of PGS methods for Mean corpuscular hemoglobin concentration. The y-axis shows the correlation (R) between predicted and observed trait levels, with error bars representing the standard error. Colours differentiate between PGS methods trained using individual-level data (IndivTune) and those trained using GWAS summary statistics (SumStatTune). Facet columns indicate the GWAS data source used for PGS derivation, including African (AFR GWAS), East Asian (EAS GWAS), European (EUR GWAS), combined African and European (EUR+AFR GWAS), and combined East Asian and European (EUR+EAS GWAS) data. Facet rows represent performance in African (AFR Target), East Asian (EAS Target), and European (EUR Target) populations.

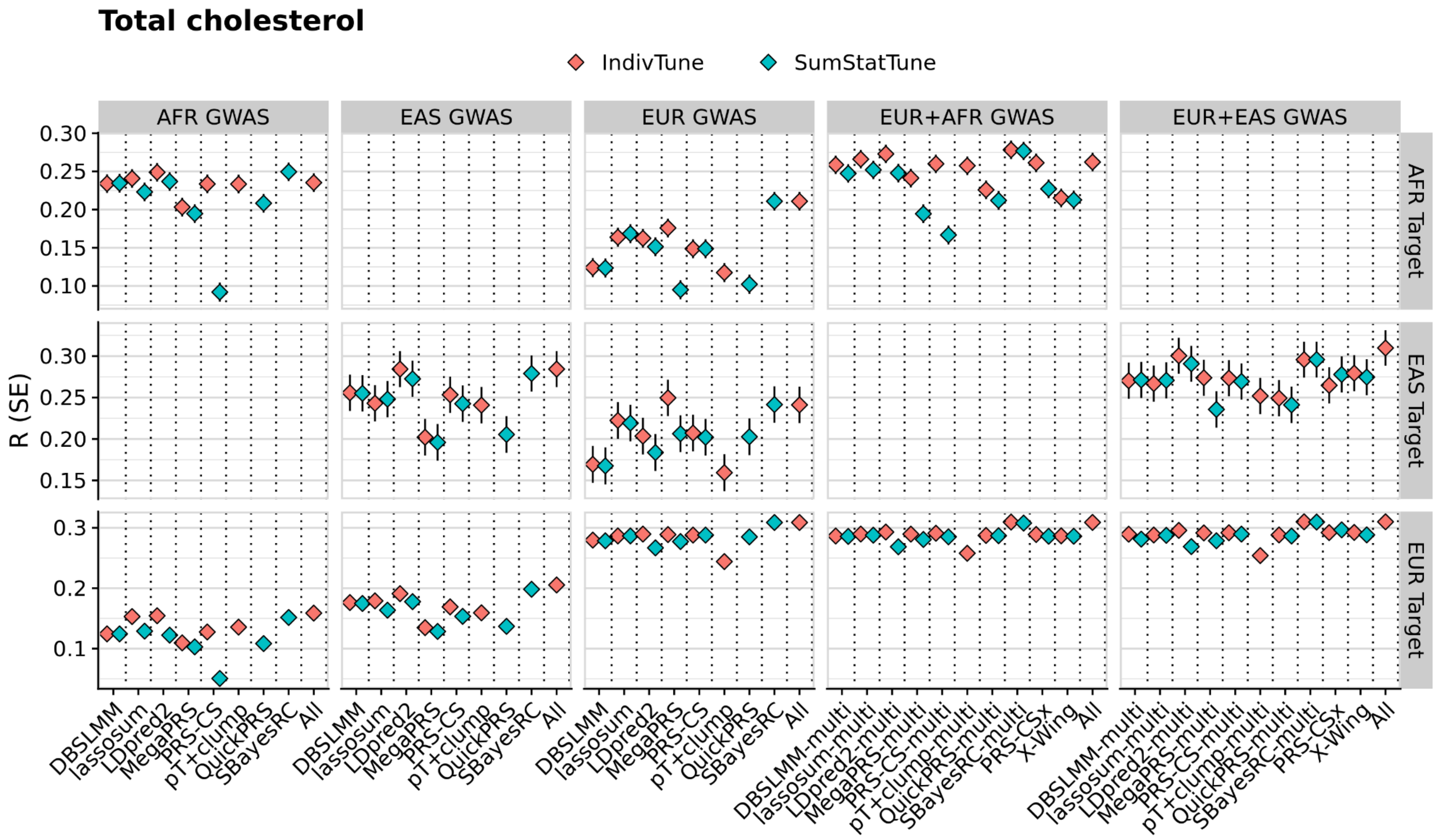

Figure S9. Predictive utility of PGS methods for Total cholesterol. The y-axis shows the correlation (R) between predicted and observed trait levels, with error bars representing the standard error. Colours differentiate between PGS methods trained using individual-level data (IndivTune) and those trained using GWAS summary statistics (SumStatTune). Facet columns indicate the GWAS data source used for PGS derivation, including African (AFR GWAS), East Asian (EAS GWAS), European (EUR GWAS), combined African and European (EUR+AFR GWAS), and combined East Asian and European (EUR+EAS GWAS) data. Facet rows represent performance in African (AFR Target), East Asian (EAS Target), and European (EUR Target) populations.

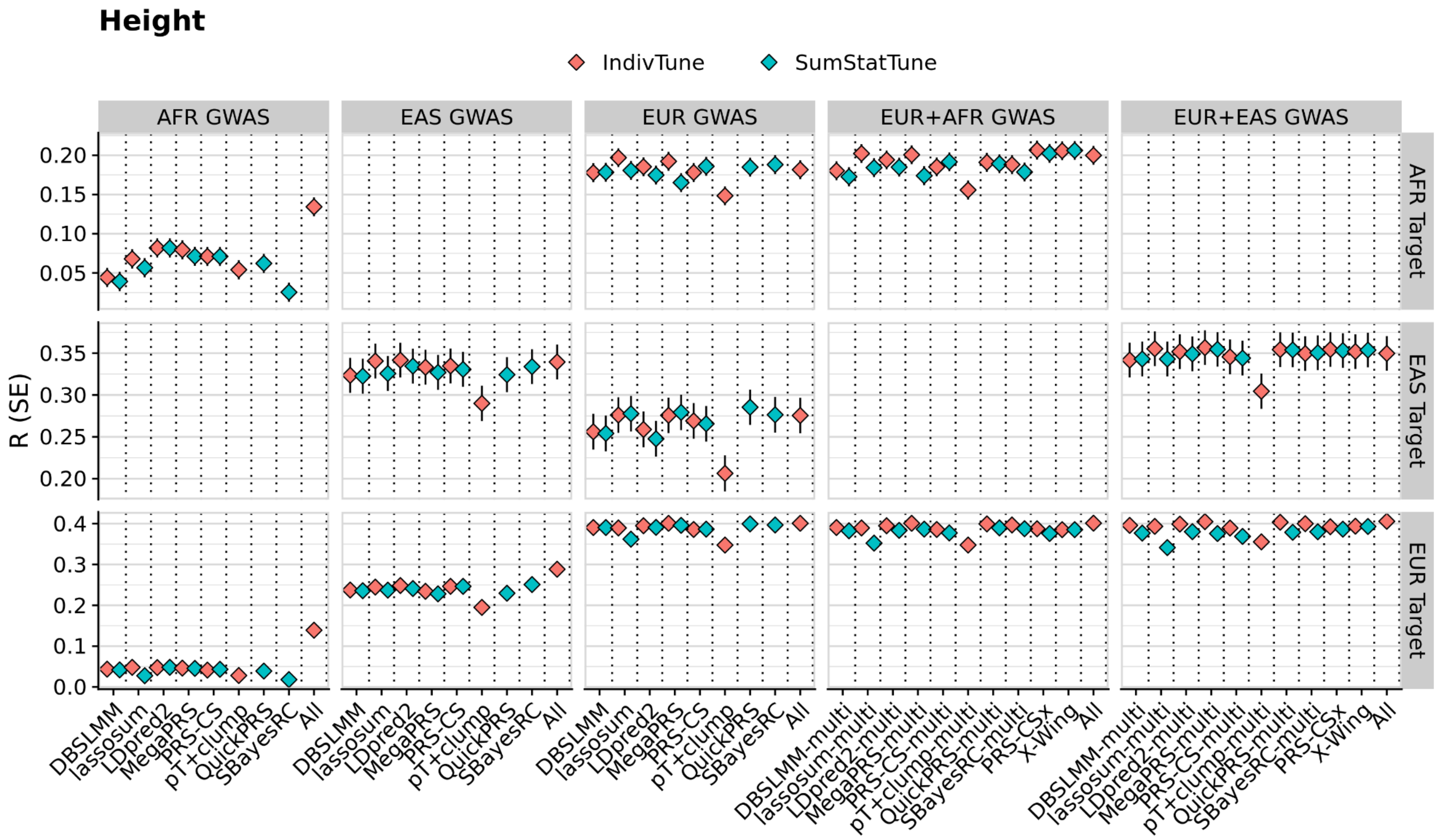

Figure S10. Predictive utility of PGS methods for Height. The y-axis shows the correlation (R) between predicted and observed trait levels, with error bars representing the standard error. Colours differentiate between PGS methods trained using individual-level data (IndivTune) and those trained using GWAS summary statistics (SumStatTune). Facet columns indicate the GWAS data source used for PGS derivation, including African (AFR GWAS), East Asian (EAS GWAS), European (EUR GWAS), combined African and European (EUR+AFR GWAS), and combined East Asian and European (EUR+EAS GWAS) data. Facet rows represent performance in African (AFR Target), East Asian (EAS Target), and European (EUR Target) populations.

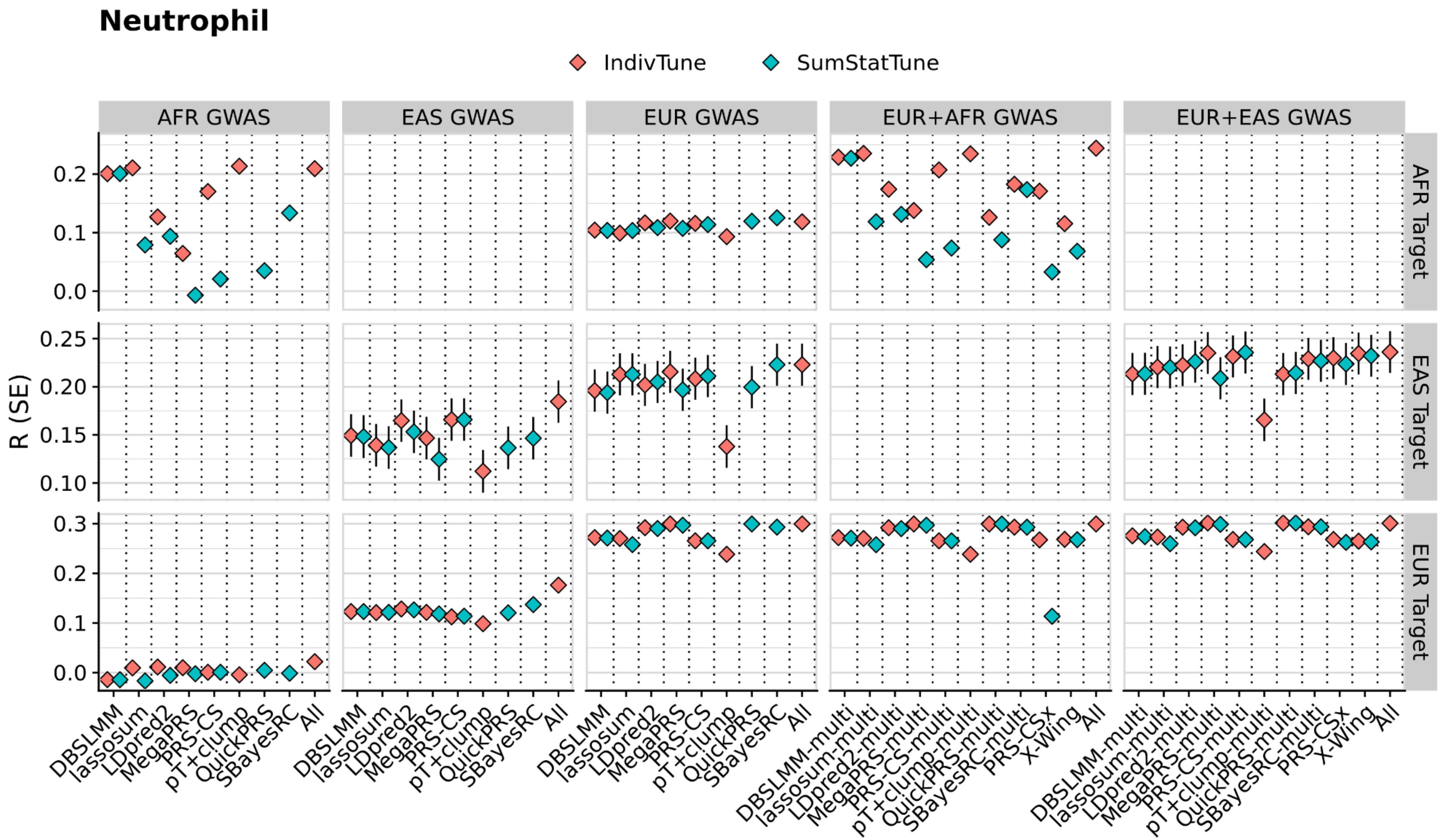

Figure S11. Predictive utility of PGS methods for Neutrophil. The y-axis shows the correlation (R) between predicted and observed trait levels, with error bars representing the standard error. Colours differentiate between PGS methods trained using individual-level data (IndivTune) and those trained using GWAS summary statistics (SumStatTune). Facet columns indicate the GWAS data source used for PGS derivation, including African (AFR GWAS), East Asian (EAS GWAS), European (EUR GWAS), combined African and European (EUR+AFR GWAS), and combined East Asian and European (EUR+EAS GWAS) data. Facet rows represent performance in African (AFR Target), East Asian (EAS Target), and European (EUR Target) populations.

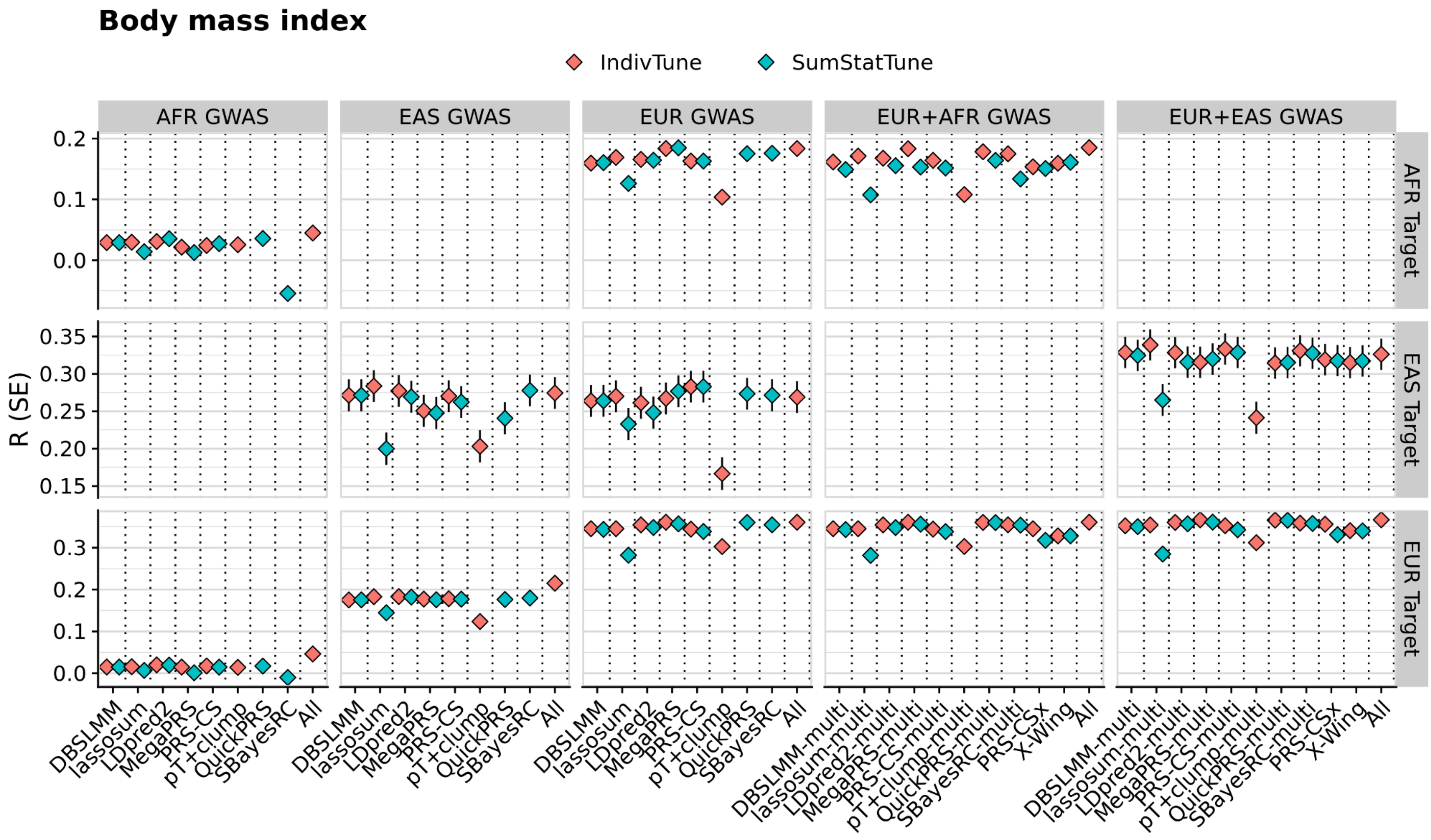

Figure S12. Predictive utility of PGS methods for Body mass index. The y-axis shows the correlation (R) between predicted and observed trait levels, with error bars representing the standard error. Colours differentiate between PGS methods trained using individual-level data (IndivTune) and those trained using GWAS summary statistics (SumStatTune). Facet columns indicate the GWAS data source used for PGS derivation, including African (AFR GWAS), East Asian (EAS GWAS), European (EUR GWAS), combined African and European (EUR+AFR GWAS), and combined East Asian and European (EUR+EAS GWAS) data. Facet rows represent performance in African (AFR Target), East Asian (EAS Target), and European (EUR Target) populations.

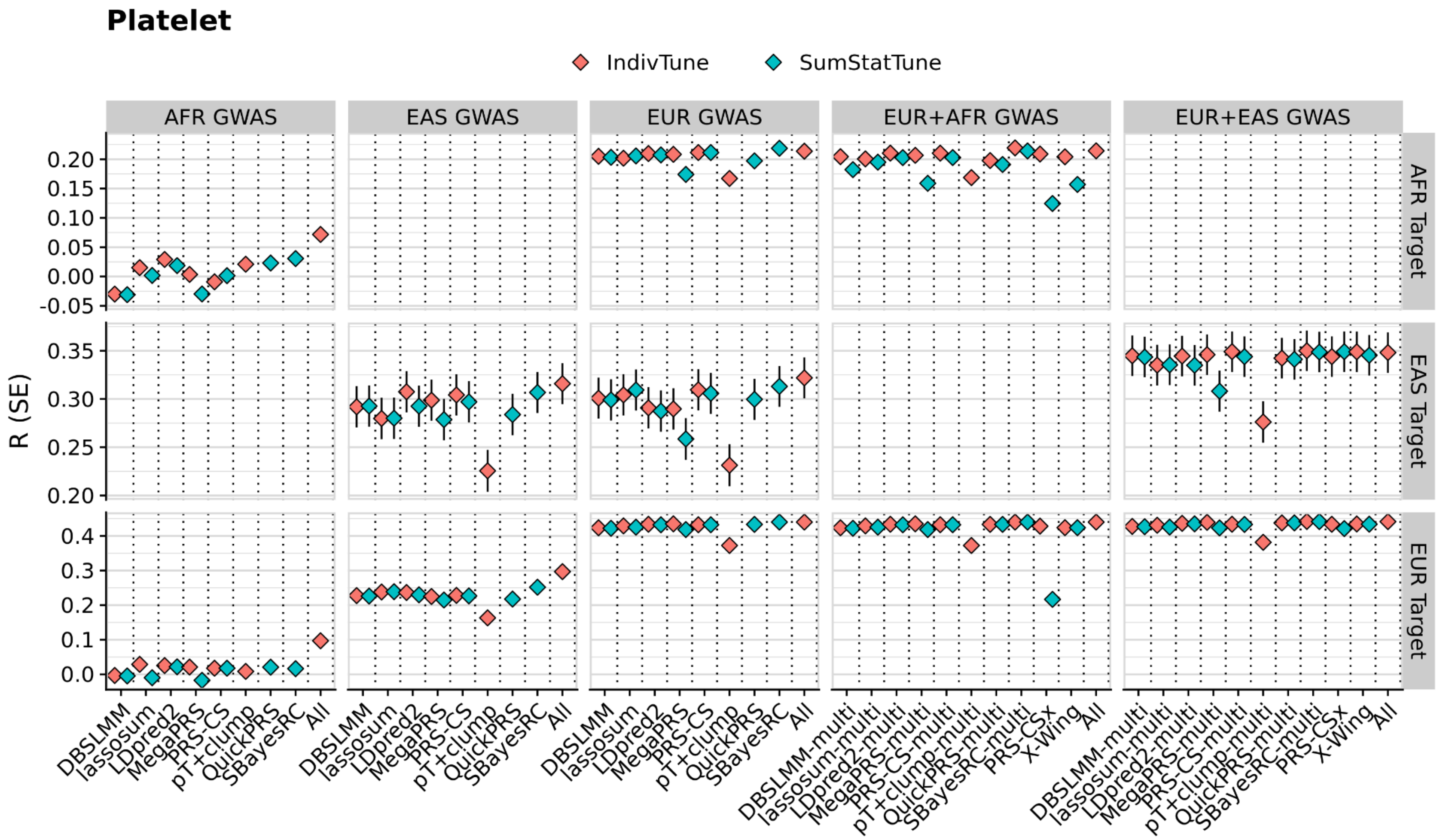

Figure S13. Predictive utility of PGS methods for Platelet. The y-axis shows the correlation (R) between predicted and observed trait levels, with error bars representing the standard error. Colours differentiate between PGS methods trained using individual-level data (IndivTune) and those trained using GWAS summary statistics (SumStatTune). Facet columns indicate the GWAS data source used for PGS derivation, including African (AFR GWAS), East Asian (EAS GWAS), European (EUR GWAS), combined African and European (EUR+AFR GWAS), and combined East Asian and European (EUR+EAS GWAS) data. Facet rows represent performance in African (AFR Target), East Asian (EAS Target), and European (EUR Target) populations.

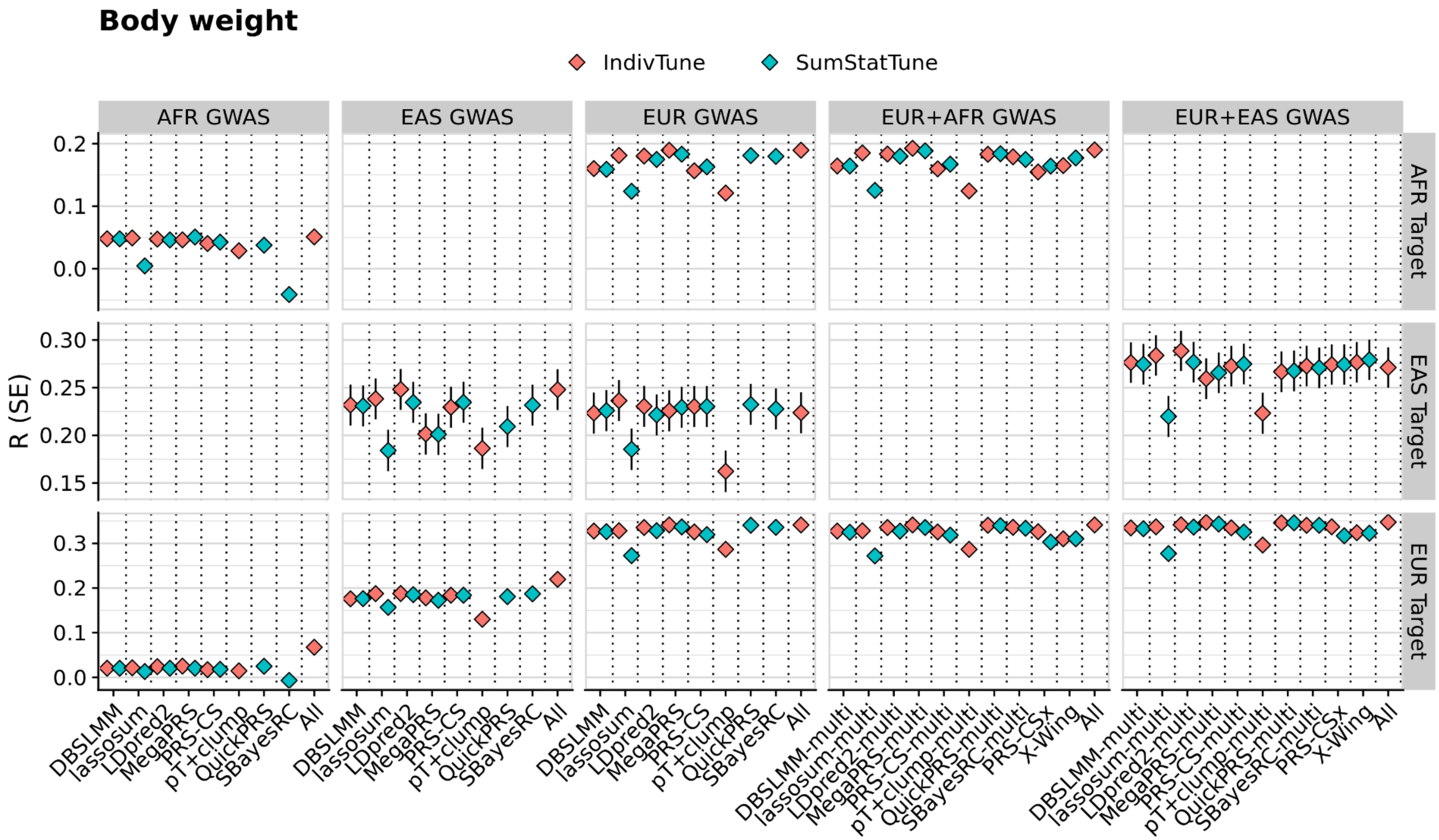

Figure S14. Predictive utility of PGS methods for Body weight. The y-axis shows the correlation (R) between predicted and observed trait levels, with error bars representing the standard error. Colours differentiate between PGS methods trained using individual-level data (IndivTune) and those trained using GWAS summary statistics (SumStatTune). Facet columns indicate the GWAS data source used for PGS derivation, including African (AFR GWAS), East Asian (EAS GWAS), European (EUR GWAS), combined African and European (EUR+AFR GWAS), and combined East Asian and European (EUR+EAS GWAS) data. Facet rows represent performance in African (AFR Target), East Asian (EAS Target), and European (EUR Target) populations.

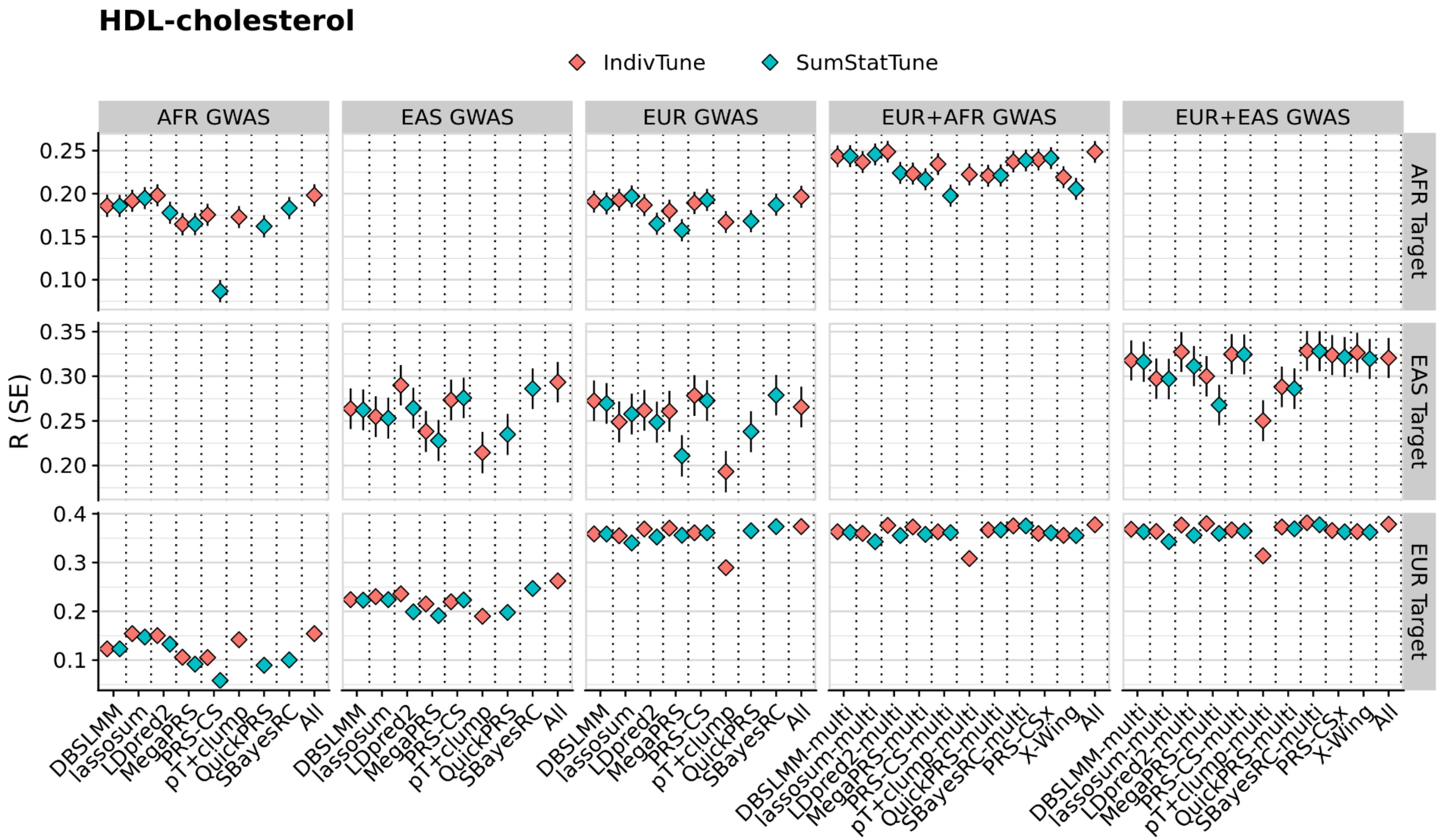

Figure S15. Predictive utility of PGS methods for HDL-cholesterol. The y-axis shows the correlation (R) between predicted and observed trait levels, with error bars representing the standard error. Colours differentiate between PGS methods trained using individual-level data (IndivTune) and those trained using GWAS summary statistics (SumStatTune). Facet columns indicate the GWAS data source used for PGS derivation, including African (AFR GWAS), East Asian (EAS GWAS), European (EUR GWAS), combined African and European (EUR+AFR GWAS), and combined East Asian and European (EUR+EAS GWAS) data. Facet rows represent performance in African (AFR Target), East Asian (EAS Target), and European (EUR Target) populations.

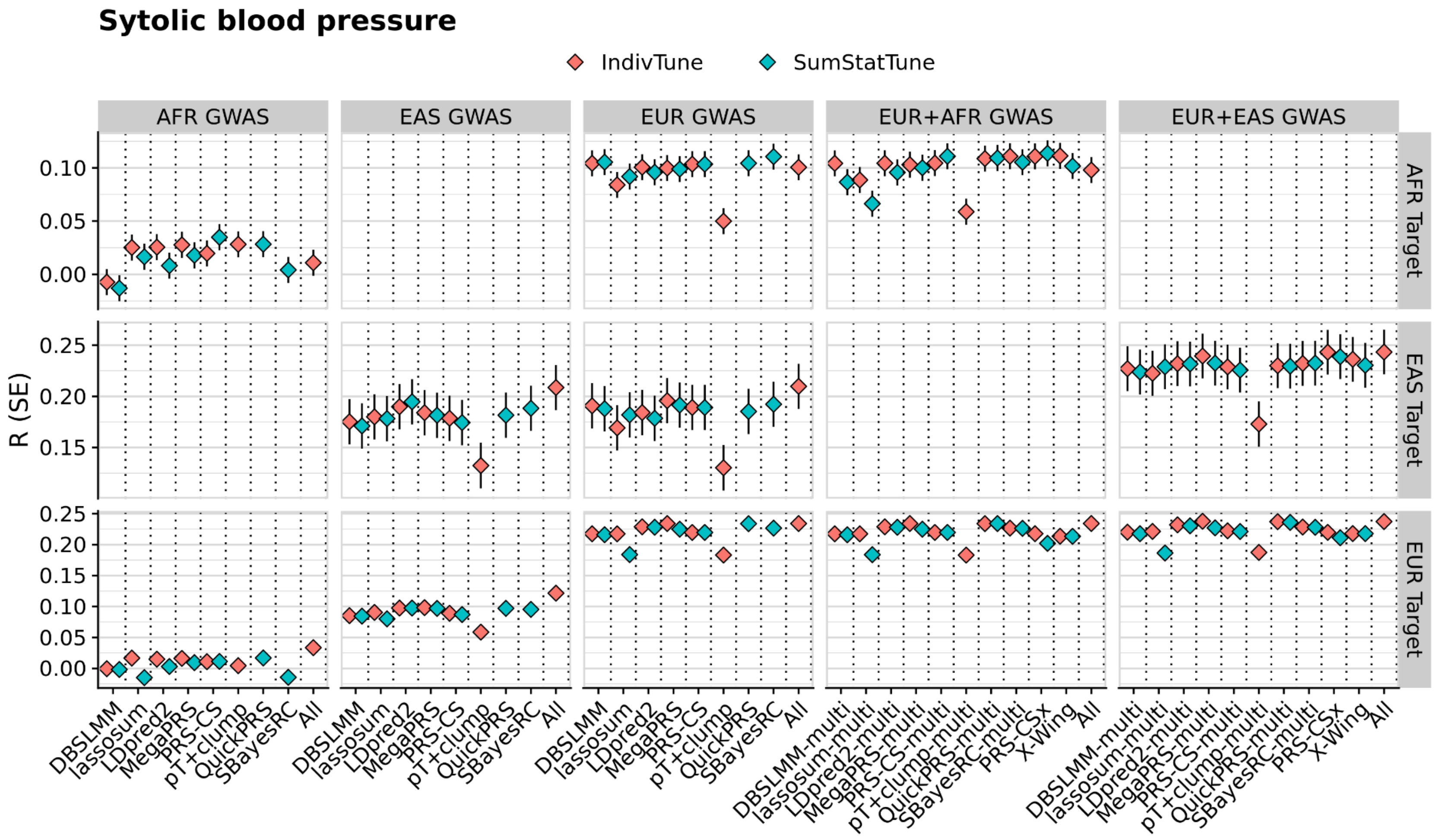

Figure S16. Predictive utility of PGS methods for Systolic blood pressure. The y-axis shows the correlation (R) between predicted and observed trait levels, with error bars representing the standard error. Colours differentiate between PGS methods trained using individual-level data (IndivTune) and those trained using GWAS summary statistics (SumStatTune). Facet columns indicate the GWAS data source used for PGS derivation, including African (AFR GWAS), East Asian (EAS GWAS), European (EUR GWAS), combined African and European (EUR+AFR GWAS), and combined East Asian and European (EUR+EAS GWAS) data. Facet rows represent performance in African (AFR Target), East Asian (EAS Target), and European (EUR Target) populations.

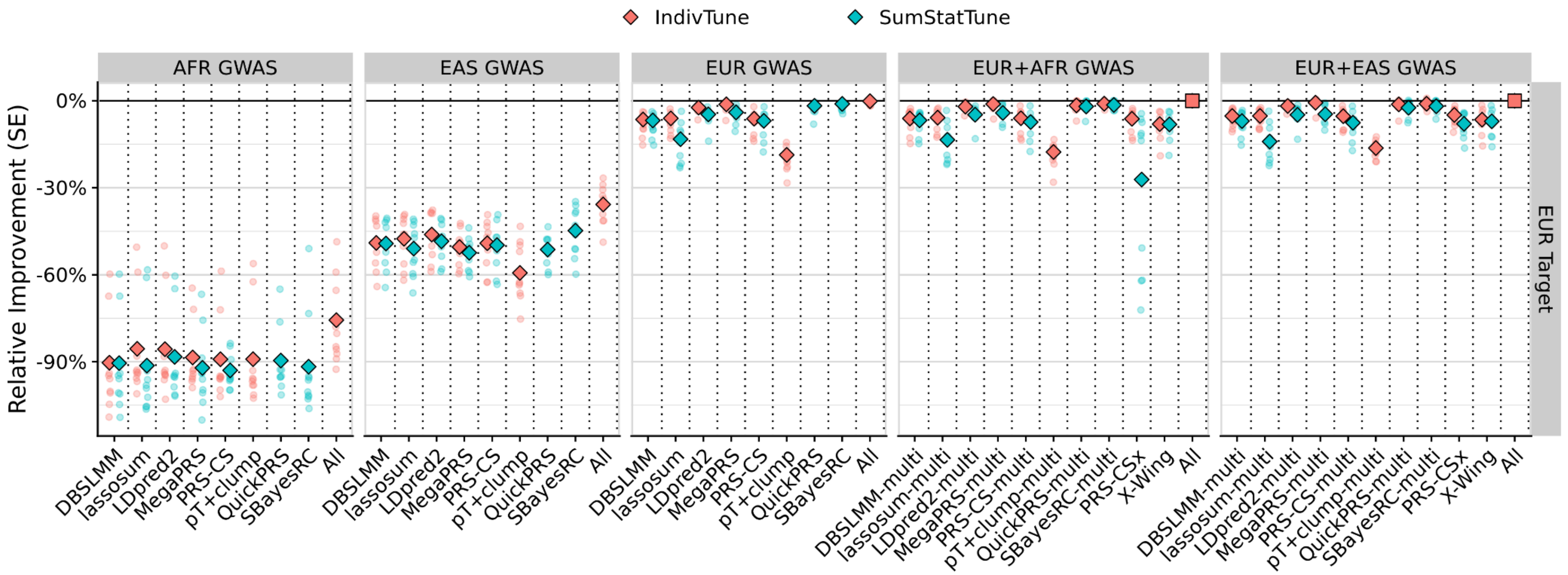

Figure S17. Relative predictive utility of PGS methods in the EUR target population. The y-axis shows the relative improvement in predictive performance compared to the multi-source 'All' model, with error bars representing the standard error. The diamond-shaped points indicate the average difference across traits, with small circular points indicating trait-specific differences. Colours indicate whether the PGS methods were trained using individual-level data (IndivTune) or GWAS summary statistics (SumStatTune). Error bars representing the standard error are plotted but are too small to be visible. Facet columns represent the source of the GWAS data used for PGS derivation, including African (AFR GWAS), East Asian (EAS GWAS), European (EUR GWAS), combined African and European (EUR+AFR GWAS), and combined East Asian and European (EUR+EAS GWAS) data. This figure demonstrates the impact of different GWAS sources and training approaches on the predictive utility of PGS in the EUR target population.

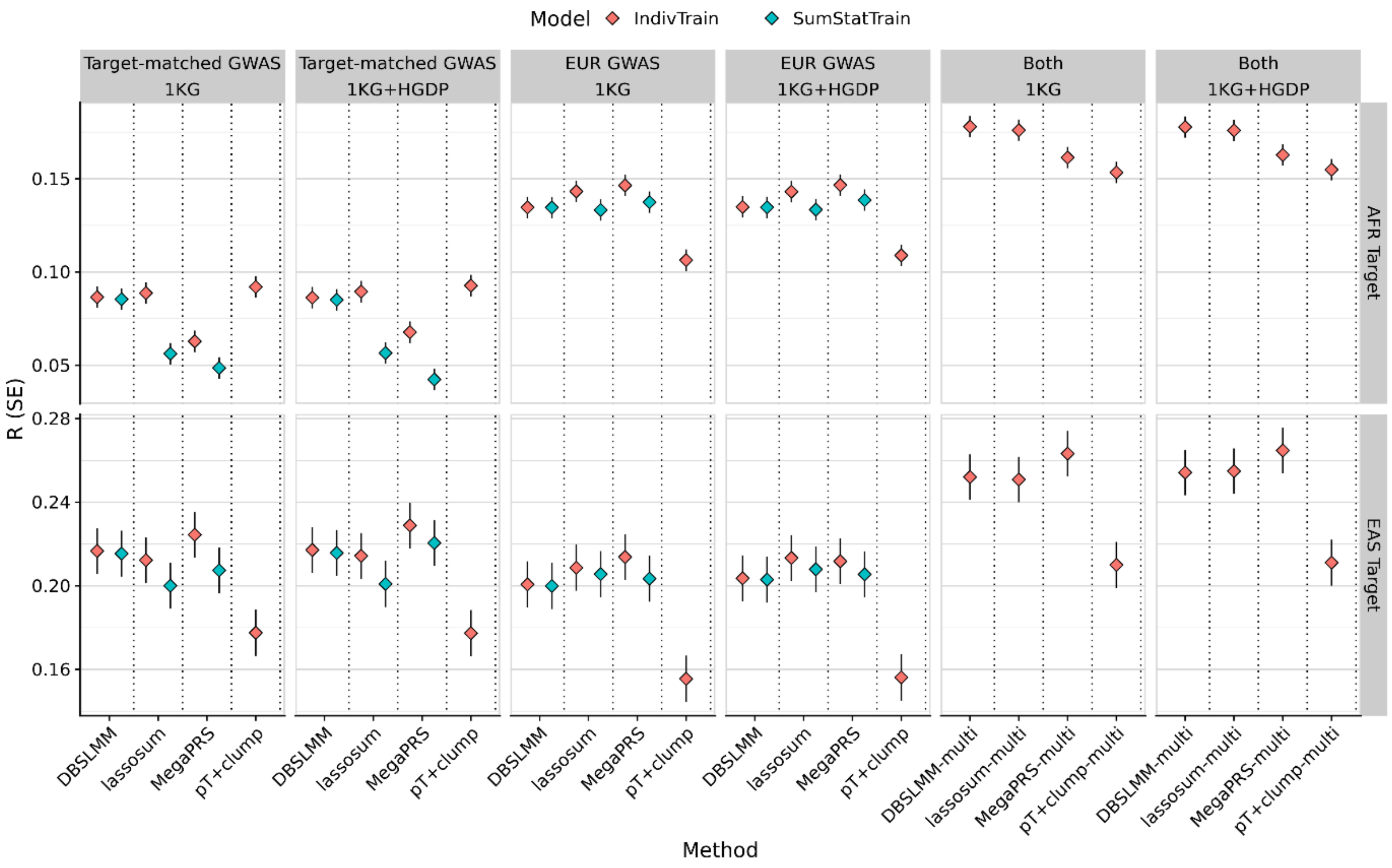

Figure S18. Comparing performance of PGS methods when using either 1KG or 1KG+HGDP reference data. The y-axis indicates the average correlation between predicted and observed values across traits, with error bars showing the standard error. Colours distinguish whether PGS methods were tuned/trained using individual-level data (IndivTrain) or GWAS summary statistics alone (SumStatTrain). 'Target-matched GWAS', 'EUR GWAS', and 'Both' facets show PGS performance using target ancestry-aligned, European, or combined GWAS data, respectively. '1KG' and '1KG+HGDP' facets show PGS performance using the 1KG or 1KG+HGDP as reference data. 'AFR Target' and 'EAS Target' facets show performance in AFR and EAS samples. There is no notable impact of the reference data on PGS performance for these methods.

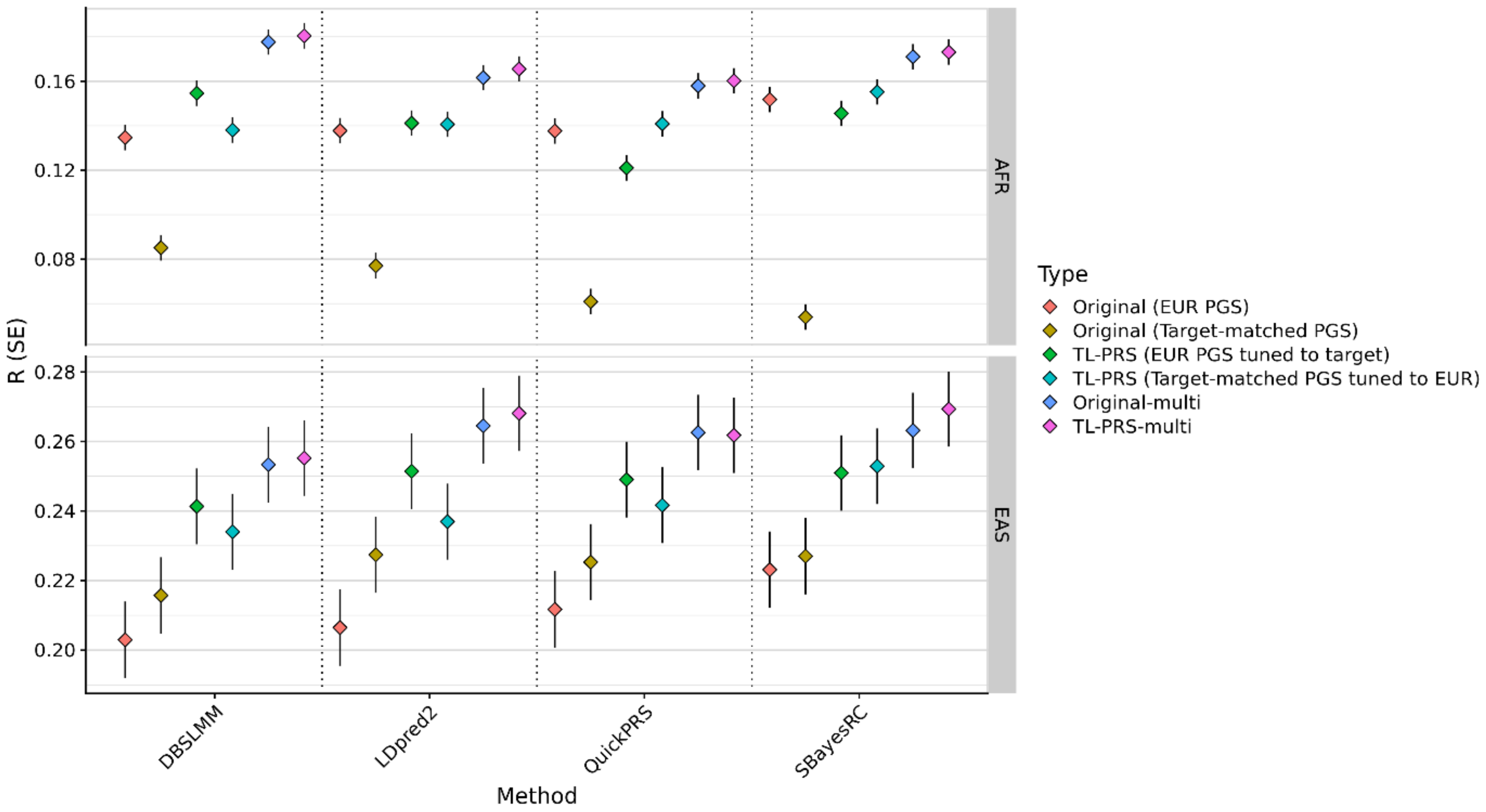

Figure S19. Comparing performance of TL-PRS adjusted PGS to unadjusted PGS. The y-axis indicates the average correlation between predicted and observed values across traits, with error bars showing the standard error. Colours distinguish different PGS models. 'Original (EUR PGS)' indicates a PGS derived using a EUR GWAS. 'Original (Target-matched PGS)' indicates a PGS derived using a target ancestry-aligned GWAS. 'TL-PRS (EUR PGS tuned to target)' indicates a EUR PGS that has been tuned towards the target population using TL-PRS. 'TL-PRS (Target-matched PGS tuned to EUR)' indicates a target ancestry-aligned PGS that has been tuned towards a EUR population using TL-PRS. 'Original-multi' indicates a model considering unadjusted PGS for both EUR and target-aligned populations, equivalent to the independently optimised multi-source approach. 'TL-PRS-multi' indicates a model considering TL-PRS-adjusted PGS for both EUR and target-aligned populations.

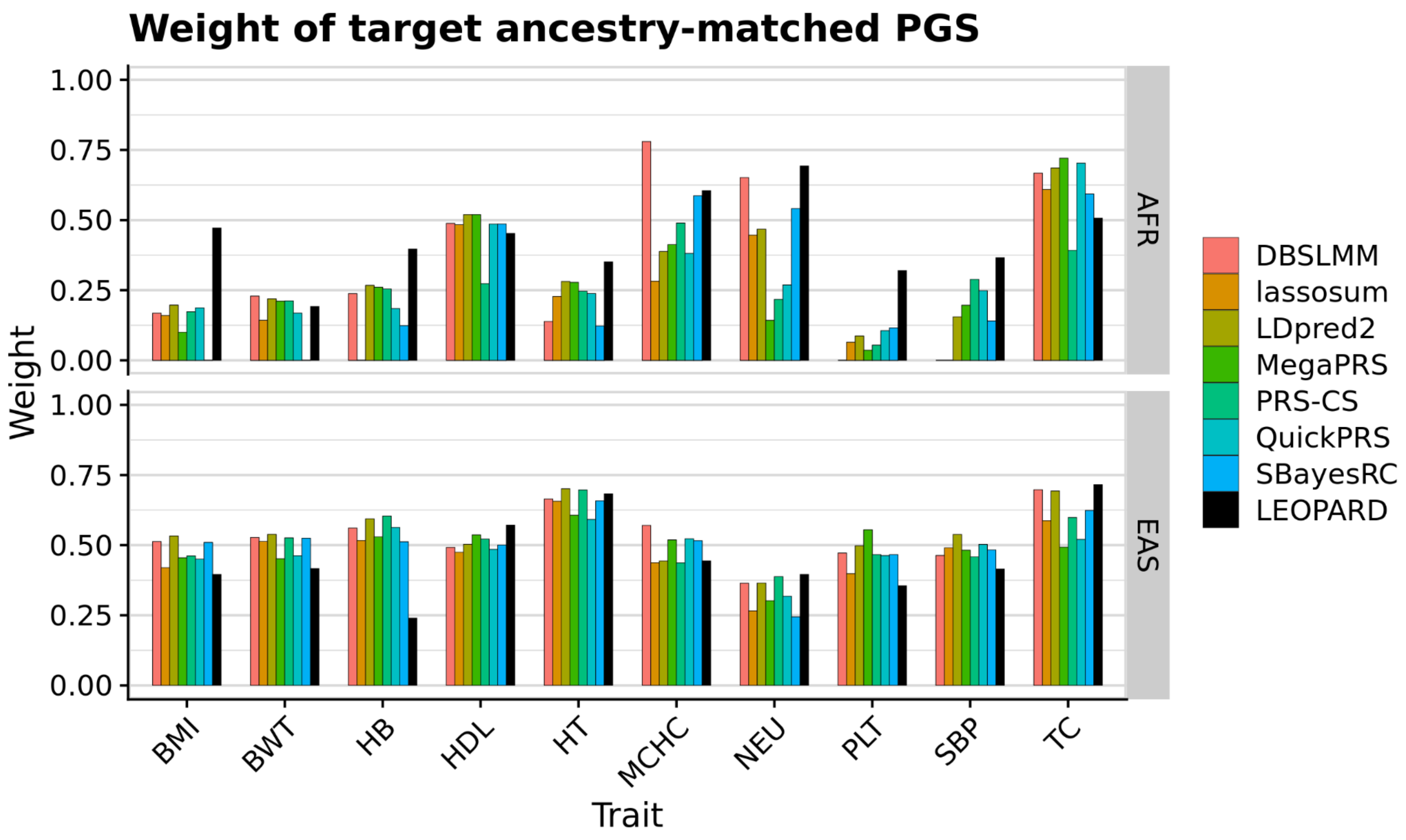

Figure S20. Observed weight of target ancestry-aligned PGS compared to weights estimated using LEOPARD (with QuickPRS). The y-axis represents the weight assigned to PGS for each method, with the x-axis showing different traits. Colours distinguish between PGS methods when estimating weights using individual-level data. The top panel shows results for the African (AFR) target population, while the bottom panel shows results for the East Asian (EAS) target population.

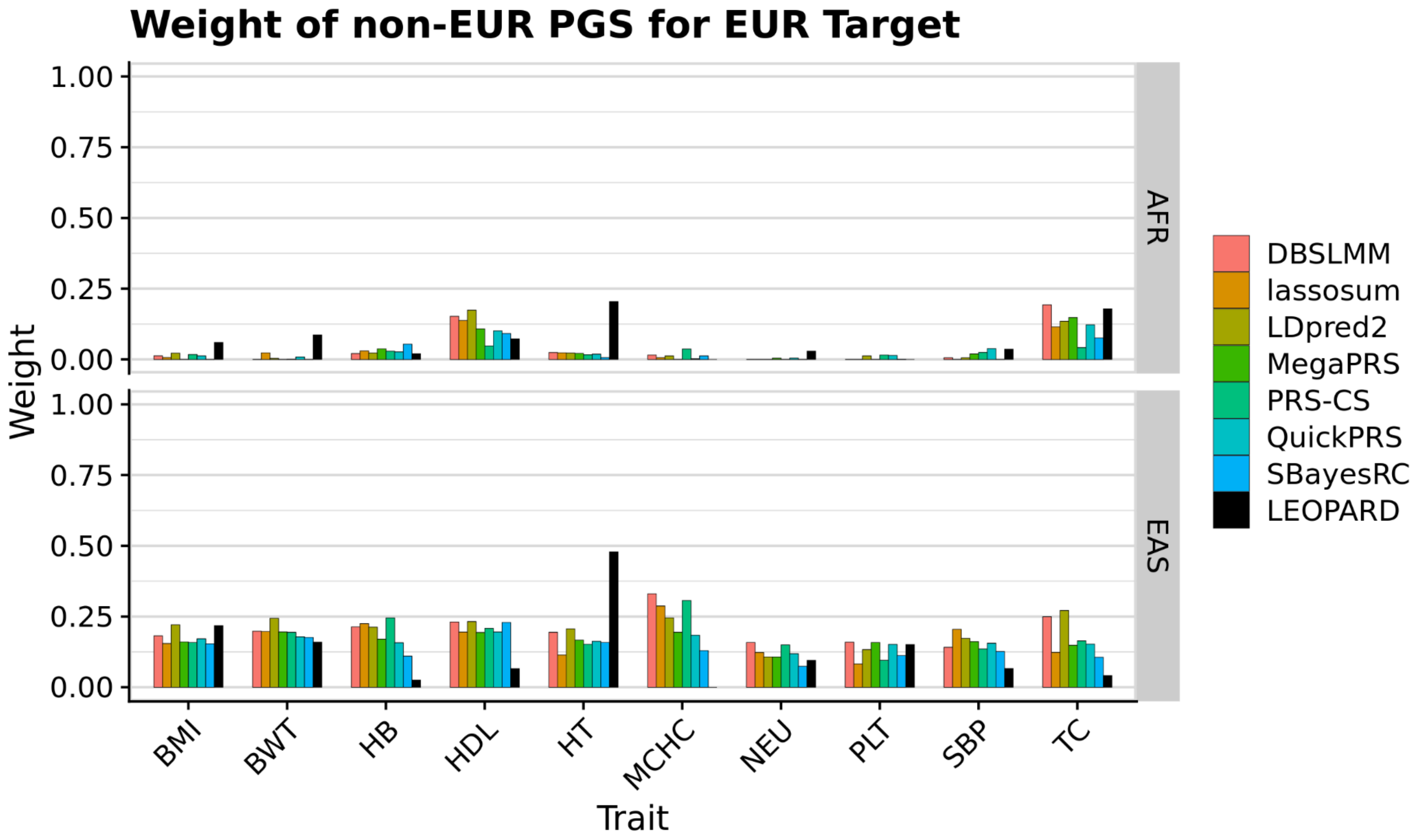

Figure S21. Observed weight of non-EUR PGS for EUR target population compared to weights estimated using LEOPARD (with QuickPRS). The y-axis represents the weight assigned to polygenic scores (PGS) for each method, with the x-axis showing different traits. Colours differentiate between PGS methods when estimating weights using individual-level data. The top panel shows results using African (AFR) GWAS in combination with European (EUR) GWAS, while the bottom panel shows results using East Asian (EAS) GWAS in combination with EUR GWAS.

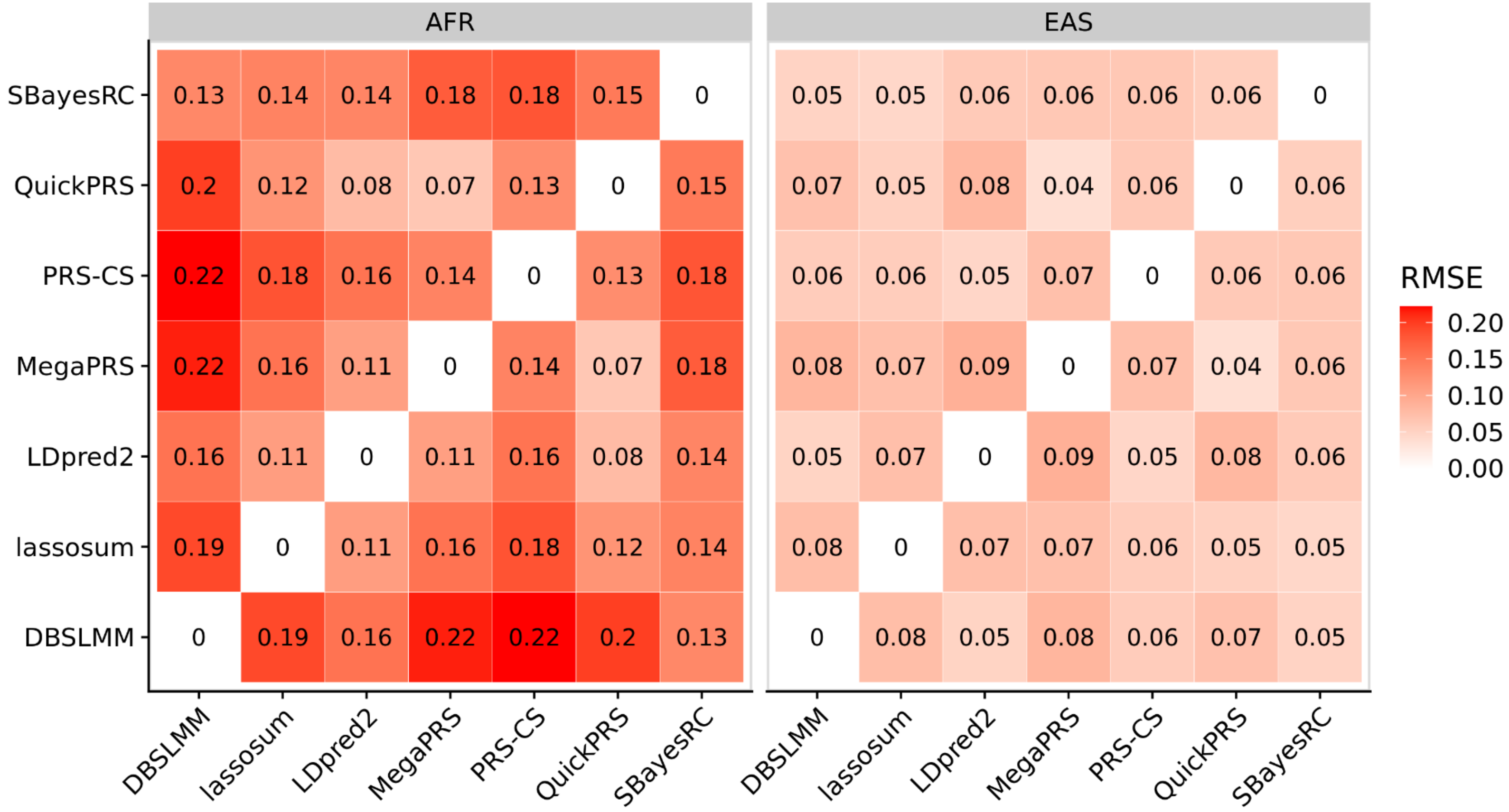

Figure S22. Root mean square error (RMSE) between observed population-specific polygenic score weights across methods. The heatmap displays RMSE values comparing the ancestry-specific weights assigned to polygenic scores by different methods, for African (AFR, left panel) and East Asian (EAS, right panel) target samples. Lower RMSE values indicate greater agreement in weight assignment between methods. Values are encoded both numerically and by colour intensity.

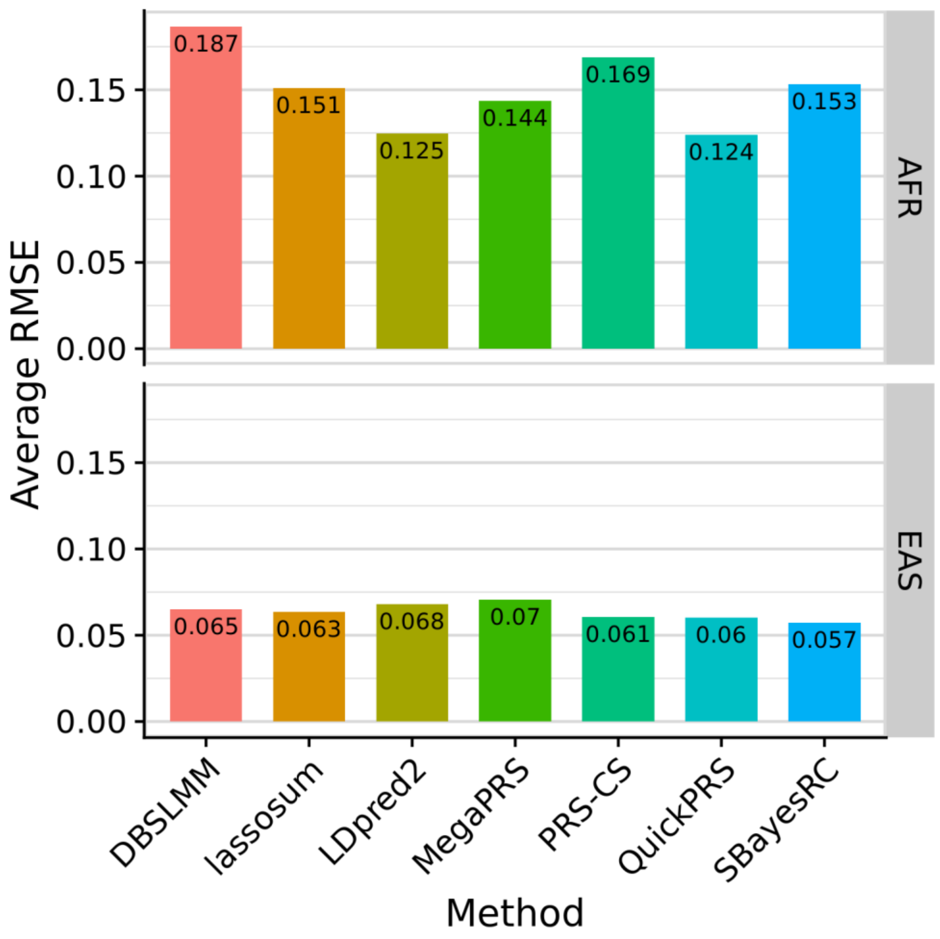

Figure S23. RMSE between observed ancestry-specific weights and LEOPARD-estimated weights across methods. Bar plots show the root mean square error (RMSE) between observed weights for ancestry-specific polygenic scores (PGS) and weights estimated using the LEOPARD method (with QuickPRS scores) for each method. Results are shown separately for African (AFR, top) and East Asian (EAS, bottom) target samples. Lower RMSE indicates better agreement with LEOPARD estimates.

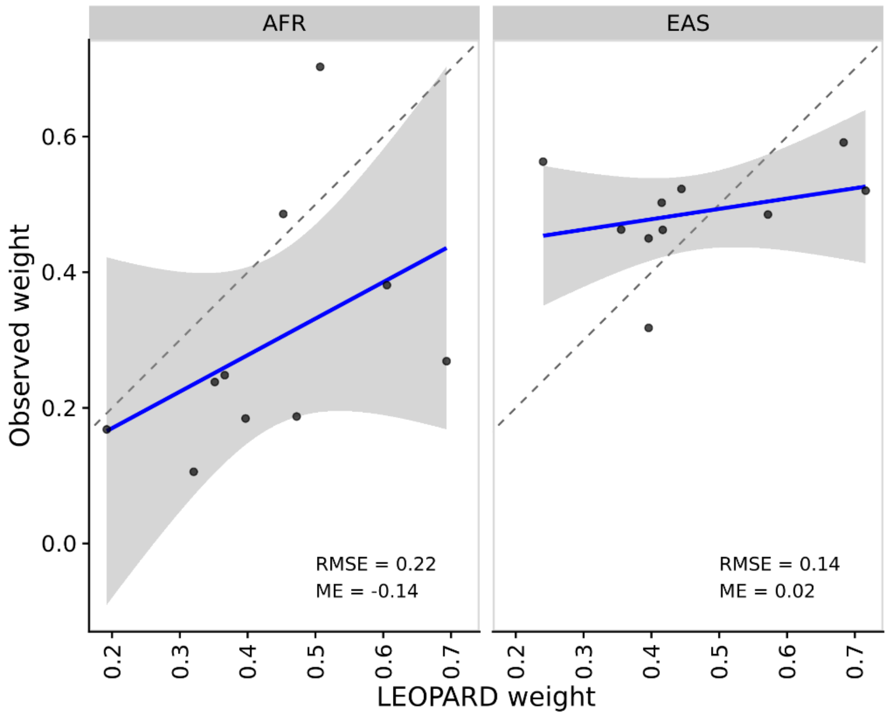

Figure S24. Calibration of LEOPARD-estimated ancestry-specific weights against observed weights derived from QuickPRS. Each point represents a trait for which ancestry-specific weights were estimated using LEOPARD (x-axis) and compared to those obtained from fitting observed QuickPRS PGS in the target sample (y-axis). Solid blue lines represent the linear fit with 95% confidence bands; dashed lines indicate the identity line (perfect calibration). RMSE and mean error (ME) are reported for each ancestry group.

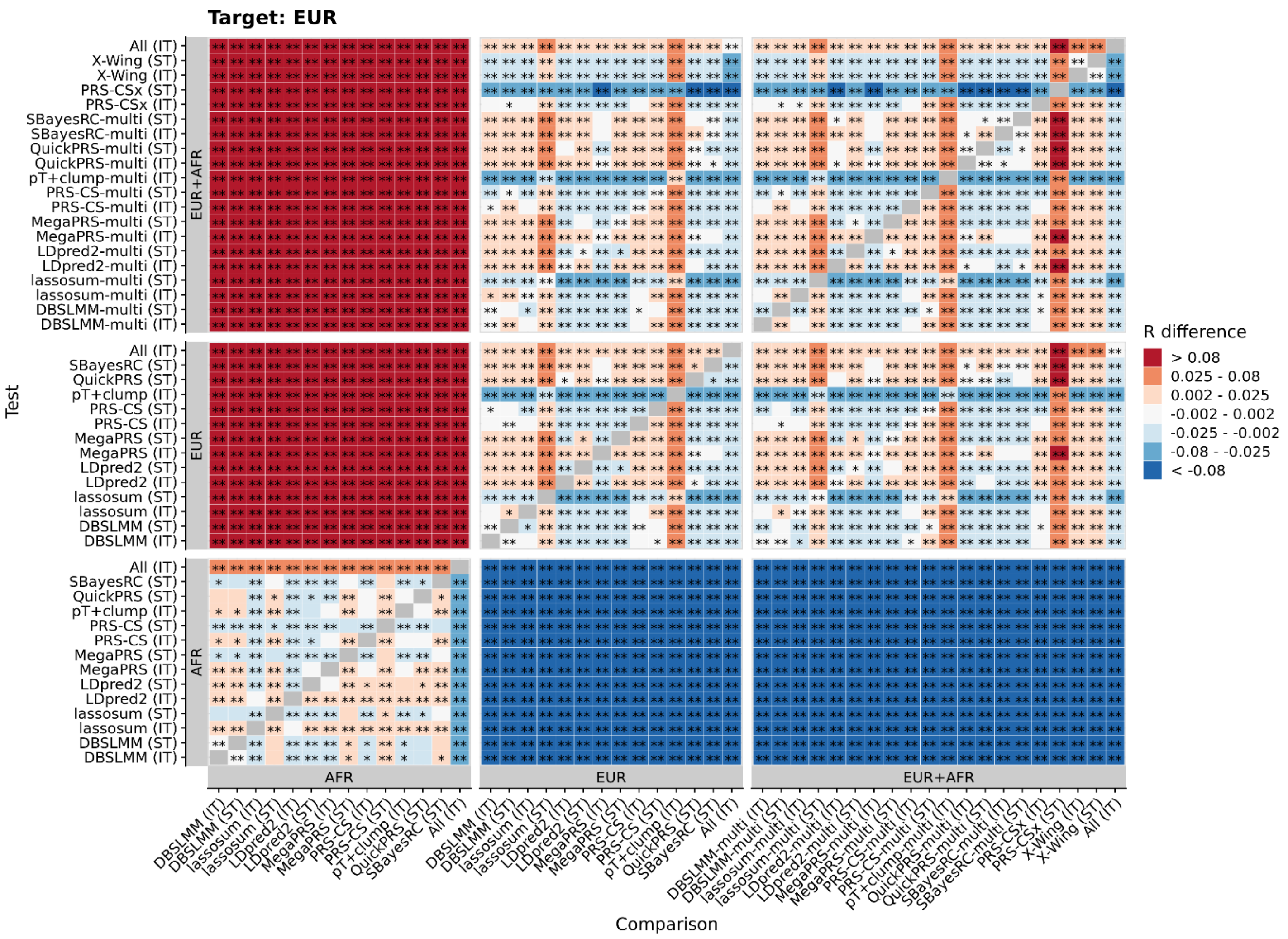

*Figure S25. Pairwise comparison between all methods in EUR target sample, using EUR and AFR GWAS data, showing average difference in observed-expected correlation. R difference = Test correlation minus Comparison correlation. Red/orange colouring indicates the Test method (shown on Y axis) performed better than the Comparison method (shown on X axis). Shows only results based on the UKB target sample when using the 1KG reference. * = p<0.05 * = p<1×10−3. P-values are two-sided. IT = IndivTune, PGS model tuned using individual-level data. ST = SumStatTune, PGS model tuned using GWAS summary statistics alone.*

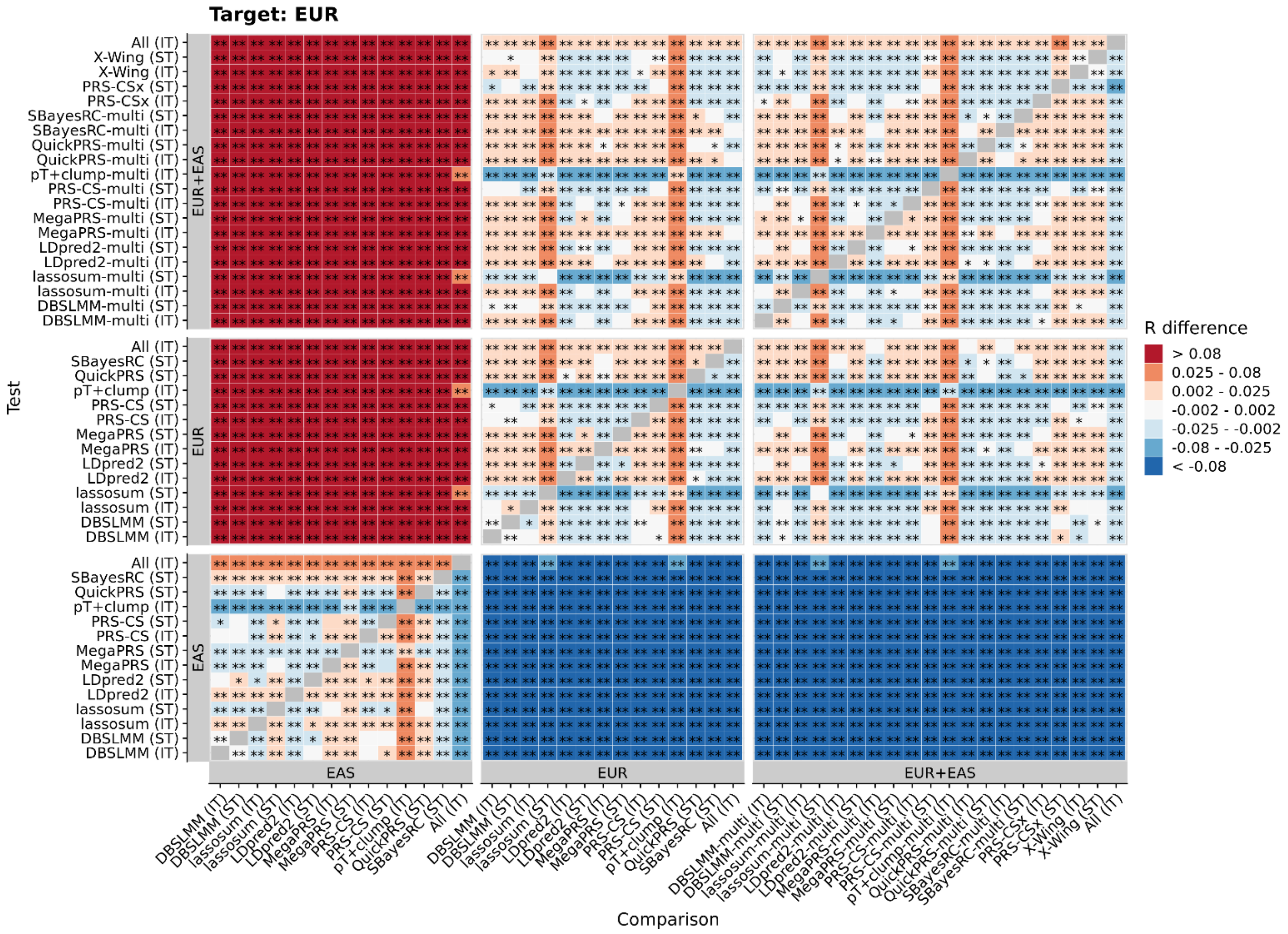

*Figure S26. Pairwise comparison between all methods in EUR target sample, using EUR and EAS GWAS data, showing average difference in observed-expected correlation. R difference = Test correlation minus Comparison correlation. Red/orange colouring indicates the Test method (shown on Y axis) performed better than the Comparison method (shown on X axis). Shows only results based on the UKB target sample when using the 1KG reference. * = p<0.05 * = p<1×10−3. P-values are two-sided. IT = IndivTune, PGS model tuned using individual-level data. ST = SumStatTune, PGS model tuned using GWAS summary statistics alone.*

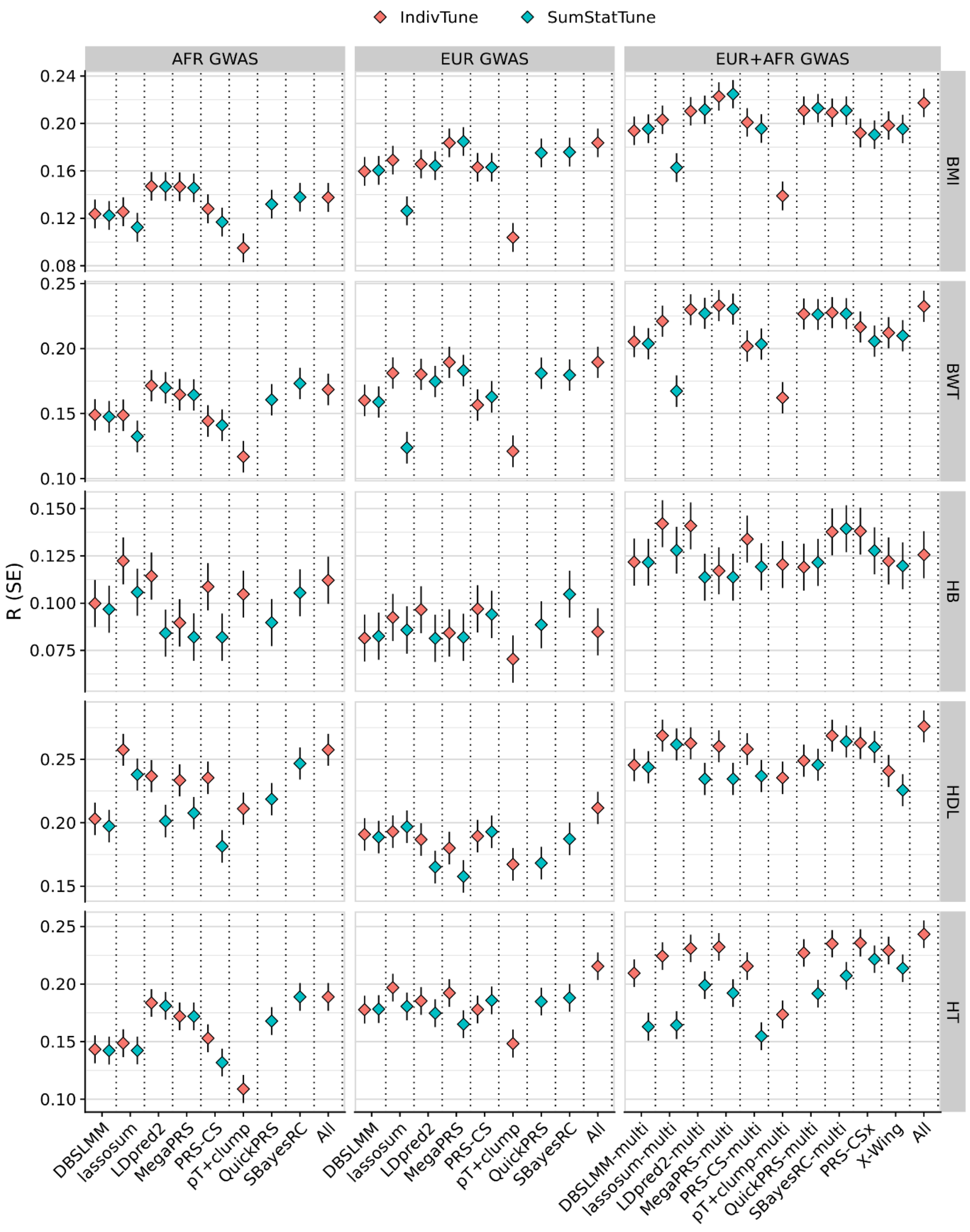

Figure S27. Trait specific predictive utility of PGS methods in AFR target sample using MVP AFR GWAS and UKB EUR GWAS. Showing results for BMI, BWT, HB, HDL, and HT. The y-axis shows the correlation (R) between predicted and observed trait levels, with error bars representing the standard error. Colours differentiate between PGS methods trained using individual-level data (IndivTune) and those trained using GWAS summary statistics (SumStatTune). Facet columns represent the source of the GWAS data used for PGS derivation, including African (AFR) GWAS, European (EUR) GWAS, and combined AFR and EUR GWAS.

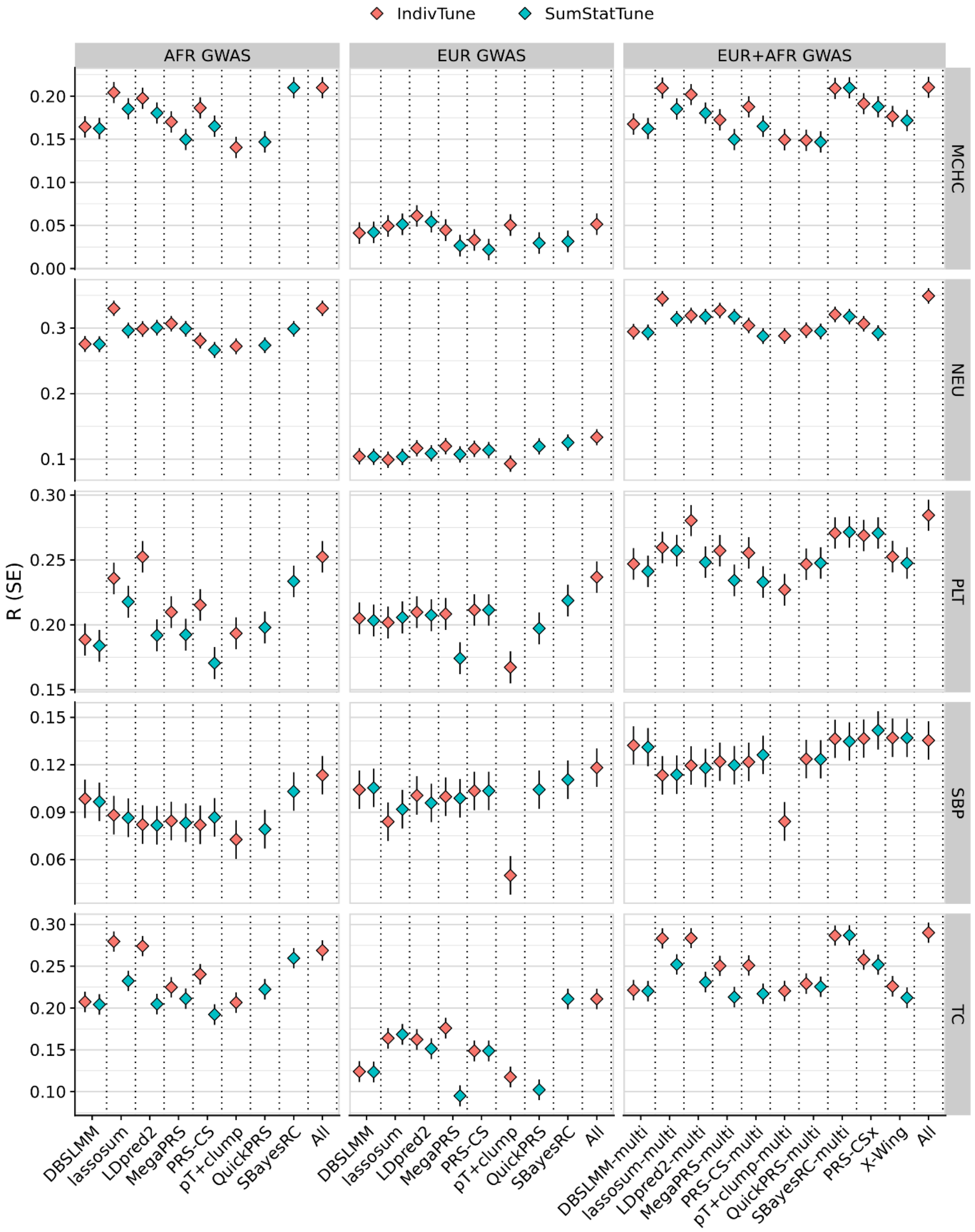

Figure S28. Trait specific predictive utility of PGS methods in AFR target sample using MVP AFR GWAS and UKB EUR GWAS. Showing results for MCHC, NEU, PLT, SBP, and TC. The y-axis shows the correlation (R) between predicted and observed trait levels, with error bars representing the standard error. Colours differentiate between PGS methods trained using individual-level data (IndivTune) and those trained using GWAS summary statistics (SumStatTune). Facet columns represent the source of the GWAS data used for PGS derivation, including African (AFR) GWAS, European (EUR) GWAS, and combined AFR and EUR GWAS.

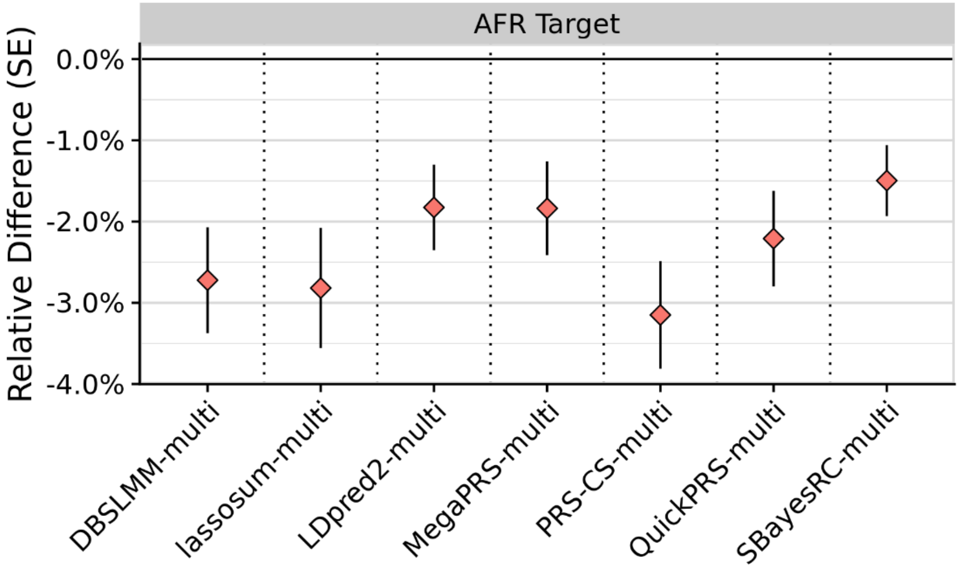

Figure S29. Comparison of prediction accuracy between SumStatTune and IndivTune PGS in the AFR target population, using LEOPARD with QuickPRS to combine population-specific scores derived from MVP (AFR) and UKB (EUR) GWAS. The y-axis shows the relative difference in correlation (R) between predicted and observed values, comparing SumStatTune PGS (using only GWAS summary statistics) to IndivTune PGS (using individual-level target data to estimate optimal weights). Error bars represent the standard error of the difference. The x-axis lists independently optimised multi-source PGS methods, where population-specific PGS were derived using a summary-statistics-only method from the corresponding single-source method (e.g., LDpred2-auto model). Negative values indicate that SumStatTune performs worse than IndivTune.

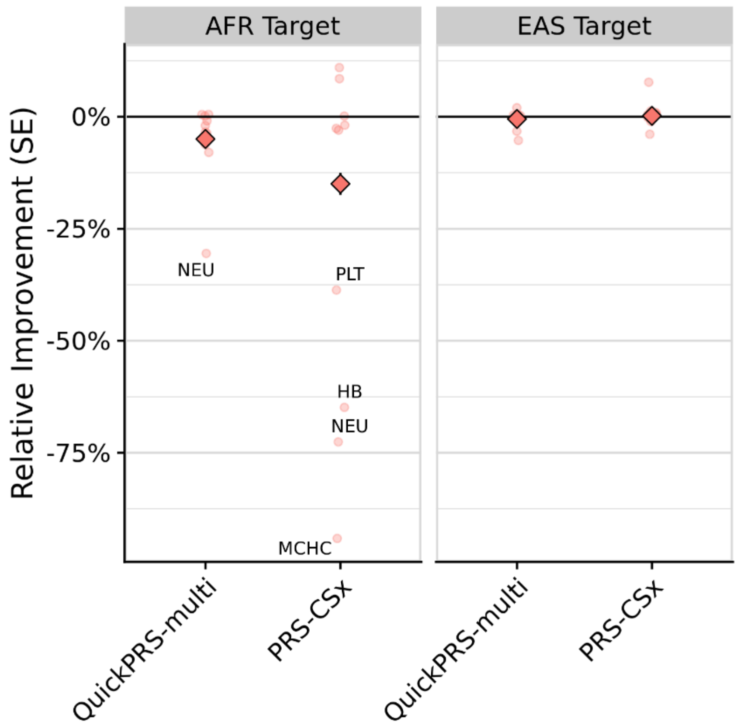

Figure S30. Comparison of prediction accuracy between SumStatTune and IndivTune approaches for combining population-specific PGS. Showing results for QuickPRS-multi (combined using LEOPARD) and PRS-CSx (combined using inverse-variance meta-analysis; --meta flag). The y-axis shows the relative difference (%) in correlation (R) between predicted and observed values when using SumStatTune compared to IndivTune approach. Each point represents a single trait, with large diamonds indicating the mean relative improvement across traits and error bars representing the standard error. Negative values indicate the IndivTune approach performed better than the SumStatTune approach. Trait names are shown for outliers with notably reduced performance. The left panel shows results for African (AFR) target samples, and the right panel shows results for East Asian (EAS) targets.

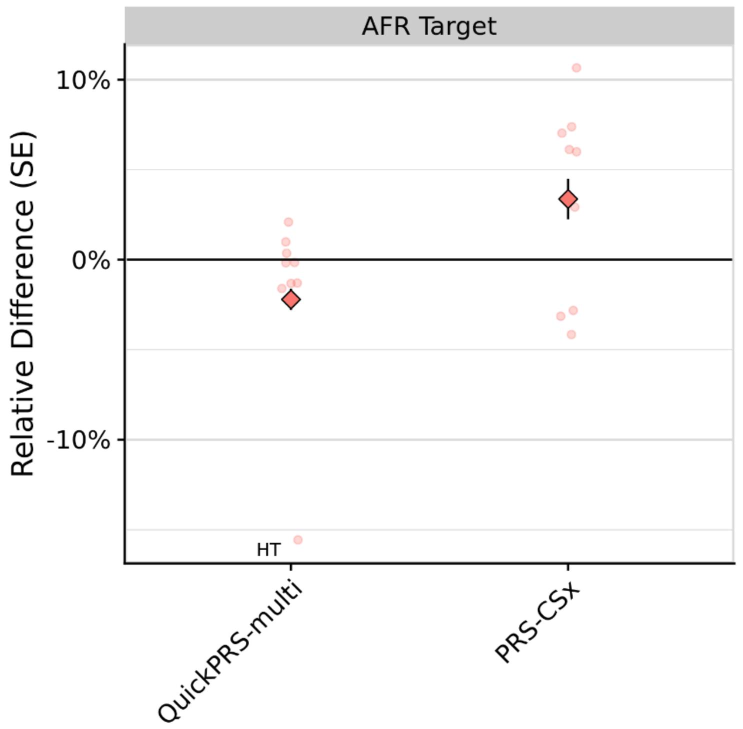

Figure S31. Comparison of prediction accuracy between SumStatTune and IndivTune approaches for combining population-specific PGS in AFR target sample using MVP AFR GWAS and UKB EUR GWAS. Showing results for QuickPRS-multi (combined using LEOPARD) and PRS-CSx (combined using inverse-variance meta-analysis; --meta flag). The y-axis shows the relative difference (%) in correlation (R) between predicted and observed values when using SumStatTune compared to IndivTune approach. Each point represents a single trait, with large diamonds indicating the mean relative improvement across traits and error bars representing the standard error. Negative values indicate the IndivTune approach performed better than the SumStatTune approach. Trait names are shown for outliers with notably reduced performance. The left panel shows results for African (AFR) target samples, and the right panel shows results for East Asian (EAS) targets.

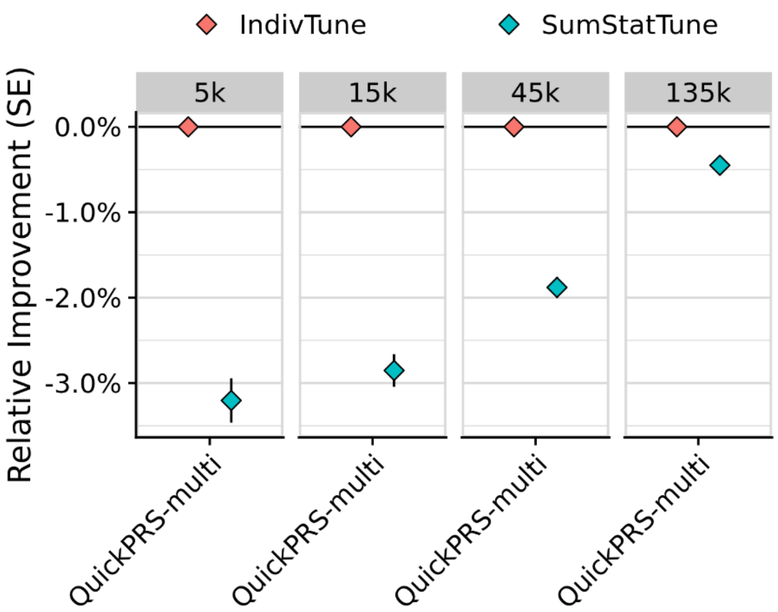

Figure S32. Relative improvement of QuickPRS-Multi using IndivTune compared to SumStatTune (using LEOPARD). Results based on EAS BBJ GWAS and downsampled EUR UKB GWAS. Facets show results across EUR UKB GWAS sample sizes.

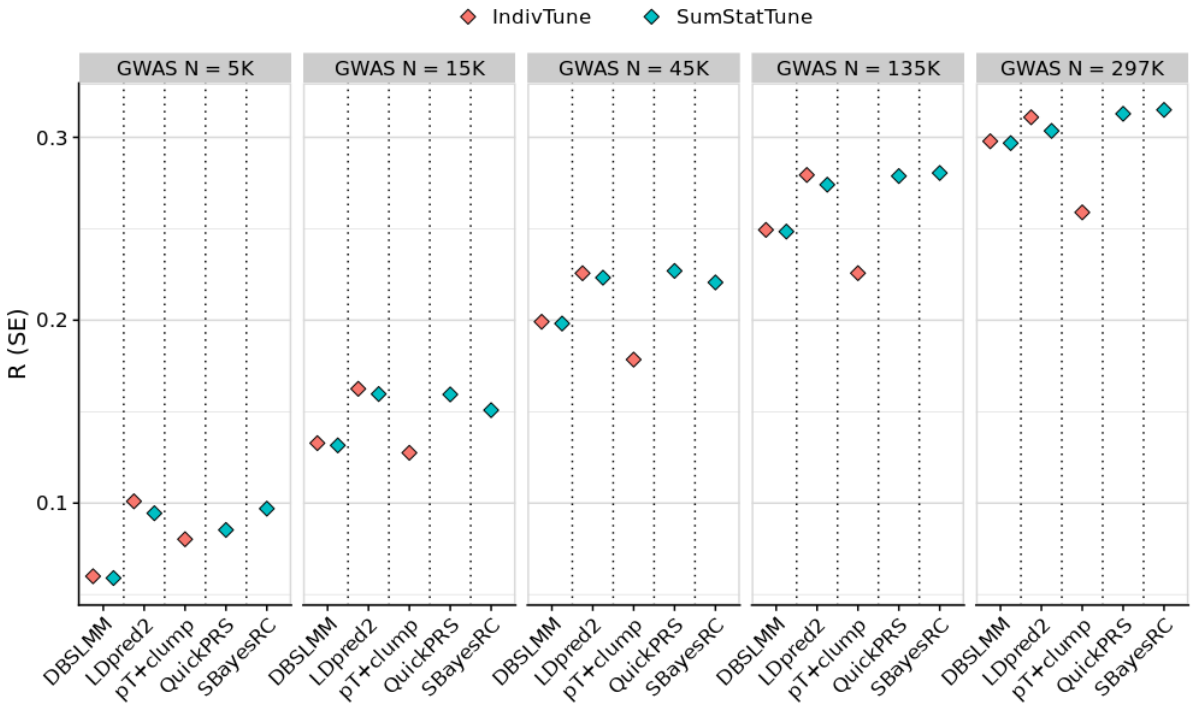

Figure S33. Average predictive performance of single-source PGS methods across downsampled EUR UKB GWAS. The y-axis shows the average correlation between observed and predicted traits in the EUR target population, with error bars representing standard errors. PGS methods were trained using downsampled UKB EUR GWAS with sample sizes of 5K, 15K, 45K, and 135K, and the full sample size of 297K. Both individual-level tuning (IndivTune; red diamonds) and summary-statistics-only tuning (SumStatTune; blue diamonds) approaches are shown.
